# Impact of Probiotic/Synbiotic Supplementation on Post-Bariatric Surgery Anthropometric and Cardiometabolic Outcomes: An Updated Systematic Review and Meta-Analysis of Randomized Controlled Trials

**DOI:** 10.3390/nu17132193

**Published:** 2025-06-30

**Authors:** Mohamed Saad Rakab, Rahma Mogahed Rateb, Alaa Maamoun, Nada Radwan, Abdalhakim Shubietah, AlMothana Manasrah, Islam Rajab, Giorgia Scichilone, Lisa Tussing-Humphreys, Abeer M. Mahmoud

**Affiliations:** 1 Faculty of Medicine, Mansoura University, Mansoura 35516, Egypt; mohamedrikab2000@gmail.com (M.S.R.); alaa.maamoun.med@gmail.com (A.M.); nadaradwan20000@gmail.com (N.R.); 2 Faculty of Medicine, Assiut University, Assiut 71111, Egypt; mogahedrateb87@gmail.com; 3 Advocate Illinois Masonic Medical Center, Chicago, IL 60657, USA; hakeemraqi@gmail.com; 4 United Health Services, Wilson Medical Center, Johnson City, NY 13790, USA; almothanamanasrah@gmail.com; 5 Department of Internal Medicine, St. Joseph’s University Medical Center, Paterson, NJ 07503, USA; islam.m.rajab@gmail.com; 6 Department of Medicine, Division of Endocrinology, Diabetes, and Metabolism, University of Illinois Chicago, Chicago, IL 60612, USA; gscic@uic.edu; 7 Department of Kinesiology and Nutrition, University of Illinois Chicago, Chicago, IL 60612, USA; tussing@uic.edu

**Keywords:** probiotics, synbiotics, bariatric surgery, gut microbiota, cardiometabolic outcomes

## Abstract

Background/Objectives: Bariatric surgery improves weight and metabolic health in individuals with severe obesity; however, challenges like gut dysbiosis and nutrient deficiencies persist postoperatively. Probiotic supplementation may enhance recovery by modulating gut microbiota. This updated meta-analysis aimed to assess the effects of probiotics/synbiotics on metabolic, anthropometric, and nutritional outcomes after bariatric surgery. Methods: A systematic review and meta-analysis of randomized controlled trials (RCTs) was conducted using PubMed, SCOPUS, Web of Science, and CENTRAL through December 2024. Studies comparing probiotics/synbiotics (which contain both probiotics and prebiotics) versus a placebo in adults post-bariatric surgery were included. Meta-analyses were conducted, with subgroup analyses by surgery type, the timing of the intervention, and probiotic formulation (PROSPERO ID: CRD420251019199). Results: Thirteen RCTs involving 809 patients were included in the analysis. Probiotic use significantly reduced BMI (MD = 0.67, 95% CI: 0.33 to 1.00), HbA1c (MD = −0.19%, 95% CI: −0.36 to −0.01), triglycerides (MD = −16.56 mg/dL), and AST levels (MD = −3.68 U/L), while increasing ALP (MD = 8.12 U/L) and vitamin D (MD = 13.68 pg/mL). Ferritin levels were significantly lower (MD = −18.89 µg/L) in the probiotic group. A subgroup analysis showed enhanced benefits in patients undergoing mini-gastric bypass, with perioperative or synbiotic interventions specifically improving triglycerides, total cholesterol, and HbA1c. Conclusions: Probiotics may offer modest but significant improvements in BMI, glycemic control, lipid profile, liver enzymes, and vitamin D levels after bariatric surgery. These findings support the potential role of probiotics/synbiotics as an adjunct therapy, though further large-scale trials are warranted to confirm long-term benefits.

## 1. Introduction

Bariatric surgery remains the most effective intervention for achieving significant and sustained weight loss in individuals with severe obesity [1]. Beyond considerable weight loss, procedures like Roux-en-Y gastric bypass and sleeve gastrectomy substantially improve glycemic control, lipid profiles, and other cardiometabolic disease risk factors [2]. However, the postoperative journey is challenging. Many patients continue to face health issues such as nutrient deficiencies, gastrointestinal discomfort, and, notably, weight regain or plateauing [3]. These persistent challenges have prompted clinicians and researchers to look into the role of the gut microbiome in long-term outcomes [4].

It is now well recognized that gut microbiota play a central role in metabolic health [5]. Changes in microbial composition influence how the body processes energy, regulates glucose, and handles inflammation [6]. Interestingly, bariatric surgery itself causes marked shifts in the gut microbiome. While some changes may enhance metabolic outcomes, others could have adverse consequences, potentially limiting the surgery’s long-term benefits [7]. This has led to growing interest in probiotic or synbiotic supplementation, a strategy aimed at repopulating the gut with beneficial bacteria to support digestion, nutrient absorption, and metabolic function [8].

Several randomized controlled trials (RCTs) have investigated the effects of probiotics in post-bariatric patients, reporting various improvements in weight loss, BMI, glucose metabolism, and lipid levels [9]. Still, the findings remain inconsistent. Although a previous meta-analysis suggested beneficial effects, differences in probiotic strains and doses, follow-up durations, and type of bariatric surgery have all contributed to a fragmented body of evidence [10]. A recent meta-umbrella review resulted in uncertainty about whether probiotics truly offer added value in enhancing post-surgical outcomes or whether the observed effects are too marginal or variable to matter in clinical practice [11].

To address this uncertainty, we conducted an updated systematic review and meta-analysis of RCTs assessing the impact of probiotic/synbiotic supplementation on anthropometric and cardiometabolic outcomes after bariatric surgery and adding a subgroup analysis to close the current gaps. This study aims to clarify the role of probiotics/synbiotics in enhancing recovery and long-term health after bariatric surgery by synthesizing findings across trials and evaluating whether the available data are sufficient to support firm conclusions.

## 2. Materials and Methods

### 2.1. Protocol Registration

Our systematic review protocol was registered in PROSPERO (registration ID: CRD420251019199). This systematic review and meta-analysis were conducted following the guidelines outlined in the Preferred Reporting Items for Systematic Reviews and Meta-Analyses (PRISMA) statement and the Cochrane Handbook for Systematic Reviews and Meta-Analysis [12,13].

### 2.2. Data Sources and Search Strategy

We systematically searched the Web of Science, SCOPUS, PubMed (MEDLINE), and Cochrane Central Register of Controlled Trials (CENTRAL) databases from their inception through December 2024 without applying any search filters. The detailed approach and results are outlined in Supplementary Table S1.

### 2.3. Eligibility Criteria

We included RCTs based on the following PICO criteria: patients were obese adults who had undergone bariatric surgery; interventions involved probiotic or synbiotic (containing both a probiotic and an adjuvant prebiotic) supplementation; comparators used matched placebo or received no supplement; and outcomes focused on anthropometric, nutritional, and cardiometabolic measures, such as body weight, BMI, glucose, insulin, vitamin B12, vitamin D, and lipid profiles. Studies were excluded if they met any of the following criteria: (1) non-human or in vitro studies; (2) overlapping or duplicate datasets; (3) book chapters, reviews, commentaries, letters to the editor, or clinical guidelines; (4) publications not available in English; and (5) studies with only prebiotic supplementation.

### 2.4. Study Selection

Search results from all the databases were imported to Rayyan [14], and duplicates were manually removed. Four authors (R.M.R, A.M., N.R., and A.S.) independently screened the remaining articles, with disagreements resolved by a fifth reviewer (M.S.R.). The screening process consisted of two stages: initial assessment of titles and abstracts to identify relevant studies, followed by full-text screening to confirm eligibility according to predefined inclusion criteria for subsequent qualitative and quantitative analyses.

### 2.5. Data Extraction

Data extraction was independently performed by four reviewers (R.M.R, A.M., N.R., and A.S.) using a standardized Excel template. Extracted information included study characteristics (country, total participants, procedure, probiotic formulation, probiotic dose, placebo details, reported outcomes of interest, and follow-up duration), baseline patient data (group sample sizes, age, sex, weight, BMI, smoking, diabetes, hypertension, dyslipidemia, hypothyroidism, fatty liver, and HbA1c levels), and clinical outcomes divided into anthropometric outcomes (BMI reduction, percentage of total weight loss, percentage of excessive weight loss, and reduction in waist circumference), lipid profile (triglycerides, cholesterol, LDL, and HDL), glycemic outcomes (HbA1c, fasting blood glucose, insulin, and homeostatic model assessment for insulin resistance (HOMA-IR)), liver enzymes (AST, ALT, GGT, and ALP), and nutritional outcomes (vitamin B12, vitamin D, ferritin, albumin, and hemoglobin). Any disagreements among reviewers were resolved through consensus discussions.

### 2.6. Risk of Bias and Certainty of Evidence

Four reviewers (R.M.R, A.M., N.R., and A.S.) independently evaluated the methodological quality of included studies using the Cochrane Risk of Bias 2 (ROB2) tool [15]. Assessments covered potential biases attributed to the randomization process, deviations from intended interventions, missing outcomes, measured outcomes, and selective reporting of results. Each outcome was assessed individually, with all decisions clearly justified and documented. Any discrepancies between reviewers were resolved through discussion and consensus.

### 2.7. Statistical Analysis

Statistical analyses were performed using R 4.4.3 software. Study results were pooled using risk ratios (RRs) for dichotomous outcomes and mean differences (MDs) for continuous outcomes, both presented with 95% confidence intervals (CIs). A random-effects model was utilized when significant heterogeneity was identified (*I*^2^ > 50% detected using the chi-square and *I*^2^ tests); otherwise, a fixed-effect model was used. Sensitivity analyses were conducted to investigate and resolve identified heterogeneity. We used the available data in the trials, and when both intention-to-treat (ITT) and per-protocol (PP) analyses were reported, we prioritized the ITT data. Median and interquartile range data were converted to means and standard deviations using the Meta-Analysis Accelerator calculator [16]. Meta-regression analysis was performed when at least ten studies reported on a specific outcome and moderator [17] using OpenMeta (Analyst) software. An omnibus *p*-value of <0.05 indicated a statistically significant association. Subgroup analyses were carried out whenever feasible. Publication bias was evaluated for primary outcomes reported by ten or more studies using funnel plots, with symmetrical distribution indicating a lower risk of publication bias [18].

## 3. Results

### 3.1. Literature Search

**Figure 1:**
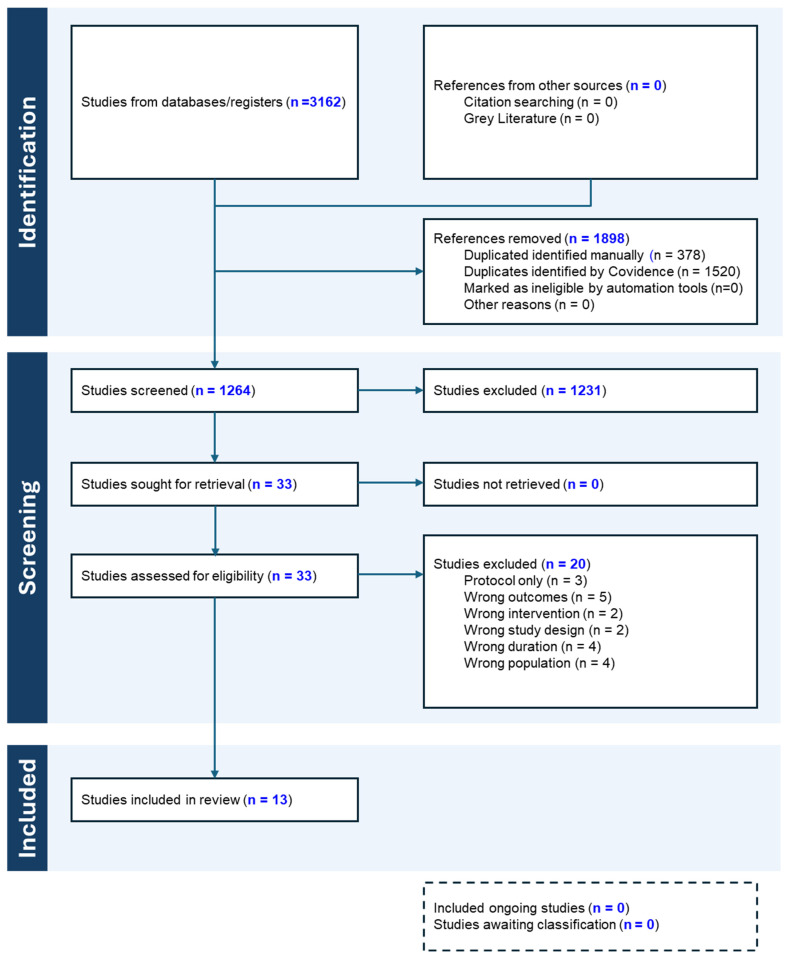
PRISMA flowchart.

A systematic search was conducted across four databases (PubMed, Scopus, Web of Science, and Cochrane Library), yielding 3162 articles, of which 1898 duplicates were excluded. After the removal of duplicates, 1264 articles underwent title and abstract screening. Of these, 33 studies qualified for full-text assessment, resulting in the inclusion of 13 RCTs. The study selection process is detailed in the PRISMA flow diagram (Figure 1).

### 3.2. Characteristics of Included Studies

Thirteen RCTs comprising 809 patients were included [19,20,21,22,23,24,25,26,27,28,29,30,31], with 401 assigned to probiotic therapy and 408 to a placebo. The studies by Chen et al. [32] and Fernandes et al. [33] were excluded for having out-of-scope outcomes and a small sample size, respectively. The included studies were conducted across seven countries: Brazil, Egypt, Germany, Iran, Israel, Poland, and the USA. The types of bariatric procedures included were Roux-en-Y gastric bypass (RYGB), laparoscopic sleeve gastrectomy (LSG), one-anastomosis gastric bypass (OAGB), and mini-gastric bypass (MGB); two studies included patients’ post-bariatric surgery without specifying the exact procedure. Most trials were single-center and had follow-up durations ranging from 1 to 6 months, with the majority having 3 months of probiotics administration postoperatively.

Probiotic interventions varied widely in composition, dose, and delivery. Most formulations contained multistrain combinations of Lactobacillus and Bifidobacterium species, with a few also including Streptococcus thermophilus or Lactococcus lactis, administered as capsules or tablets. Placebos included inert starch-based capsules, manipulated tablets, or calcium/micronutrient preparations. Full study-level characteristics and participant demographics are detailed in Table 1 and Table 2.

### 3.3. Risk of Bias Assessment

All the included studies showed a low risk of bias except for some concerns of potential deviations from intended interventions in Kazzi et al. (2021) [27], as the study was single-blinded. Sherf-Dagan (2018) [29] was judged as high-risk due to the high possibility of missing data (Supplementary Figure S1).

### 3.4. Anthropometric Outcomes

**Figure 2:**
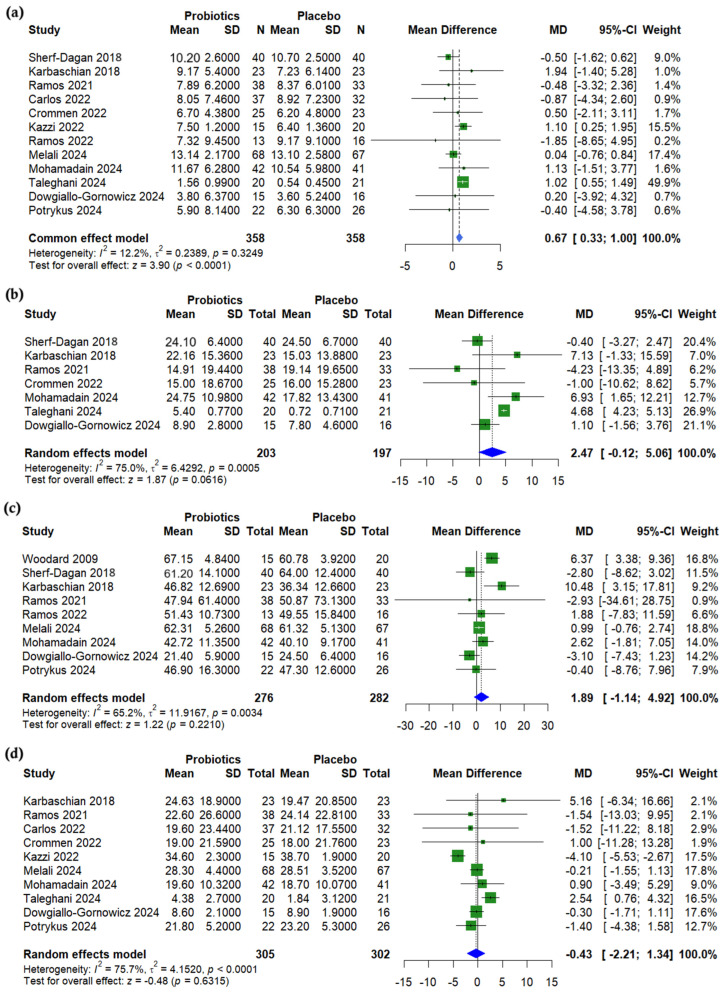
Forest plots of anthropometric measurements: (**a**) BMI reduction, kg/m^2^, (**b**) Waist circumference (WC) reduction, cm, (**c**) Excessive weight loss % (EWL%), (**d**) Total weight loss % (TWL%). [19,20,21,22,23,24,25,26,27,28,29,30,31].

A pooled analysis of body mass index (BMI) showed a significant reduction in the probiotics group compared to a placebo (with a mean difference (MD) of 0.67, 95% CI (0.33 to 1.00), *p* < 0.001, *I*^2^ = 12%) (Figure 2a). On the other hand, no significant differences were observed between groups for waist circumference (MD = 2.47, 95% CI (−0.12 to 5.06), *p* = 0.062, *I*^2^ = 75.0%) (Figure 2b), % excess weight loss (%EWL) (MD = 1.89, 95% CI (−1.14 to 4.92), *p* = 0.221, *I*^2^ = 65.2%) (Figure 2c), or % total weight loss (%TWL) (MD = −0.43, 95% CI (−2.21 to 1.34), *p* = 0.632, *I*^2^ = 75.7%) (Figure 2d). A leave-one-out sensitivity analysis showed that the exclusion of Ghafouri-Taleghani (2024) [20] reduced heterogeneity in waist circumference change (*I*^2^ = 45.4%, *p* = 0.103) (Supplementary Figure S2). The exclusion of Kazzi (2021) [27] notably reduced the heterogeneity of the %TWL (*I*^2^ = 35.4%, *p* = 0.135) (Supplementary Figure S3).

A meta-regression showed no significant association between age and BMI change (*p* = 0.244) (Supplementary Figure S4). However, a higher baseline BMI was significantly associated with smaller probiotic-related BMI reductions (*β* = −0.029, *p* = 0.010) (Supplementary Figure S5). Similarly, the meta-regression analysis revealed a non-significant trend between age %TWL (*p* = 0.082) (Supplementary Figure S6), while a higher baseline BMI was significantly associated with a reduced %TWL (*β* = −0.178, *p* = 0.009) (Supplementary Figure S7). The funnel plot for BMI was asymmetrical, suggesting potential publication bias, with the underrepresentation of smaller studies showing adverse or null effects (Supplementary Figure S8). Meanwhile, the funnel plot for TWL appears symmetrical, indicating no evidence of publication bias among the included studies (Supplementary Figure S9).

### 3.5. Lipid Profile

**Figure 3:**
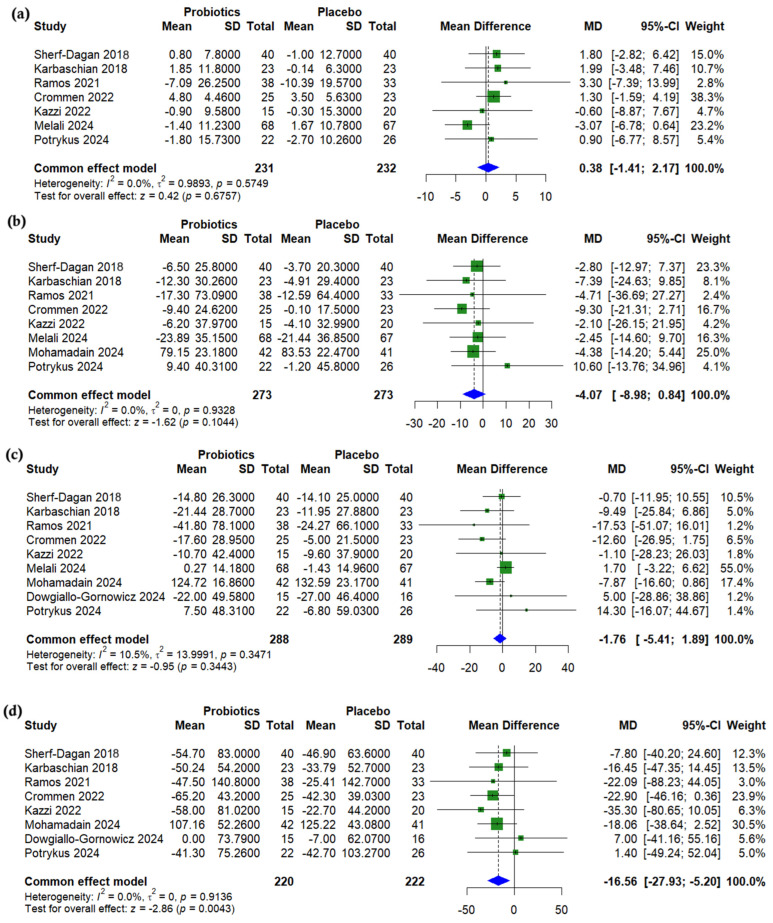
Forest plots of lipid profile (change from baseline): (**a**) HDL, mg/dL, (**b**) LDL, mg/dL, (**c**) Total cholesterol (TC), mg/dL, (**d**) Triglycerides, mg/dL [19,22,23,24,25,26,27,29,31].

A pooled analysis showed no significant differences between probiotics and a placebo after bariatric surgery for HDL cholesterol (MD = 0.38, 95% CI (−1.41 to 2.17), *p* = 0.676, *I*^2^ = 0%) (Figure 3a), LDL cholesterol (MD = −4.07, 95% CI (−8.98 to 0.84), *p* = 0.104, *I*^2^ = 0%) (Figure 3b), or total cholesterol (MD = −1.76, 95% CI (−5.41 to 1.89), *p* = 0.344, *I*^2^ = 10.5%) (Figure 3c). However, triglycerides were significantly lower in the probiotics group (MD = −16.56, 95% CI (−27.93 to −5.20), *p* = 0.004, *I*^2^ = 0%) (Figure 3d).

### 3.6. Glycemic Outcomes

**Figure 4:**
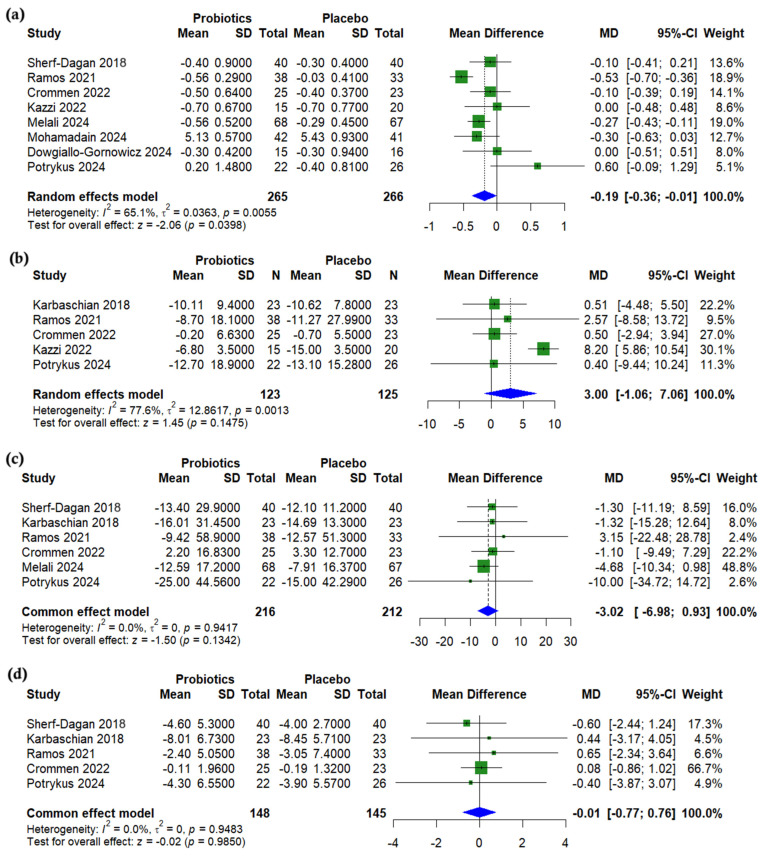
Forest plots of glycemic outcomes (change from baseline): (**a**) HbA1c, %, (**b**) Insulin, µU/mL, (**c**) Fasting blood glucose, mg/dL, (**d**) HOMA-IR, unitless [19,22,23,24,25,26,27,29,31].

A pooled analysis showed a significant reduction in HbA1c in the probiotics group compared to a placebo after bariatric surgery (MD = −0.19%, 95% CI (−0.36 to −0.01), *p* = 0.0398, *I*^2^ = 65.1%) (Figure 4a). The level of fasting insulin was non-significantly higher with probiotics (MD = 3.00 µU/mL, 95% CI (−1.06 to 7.06), *p* = 0.1475, *I*^2^ = 77.6%) (Figure 4b). No significant differences were found for fasting blood glucose (MD = −3.02 mg/dL, 95% CI (−6.98 to 0.93), *p* = 0.134, *I*^2^ = 0%) (Figure 4c) or HOMA-IR (MD = −0.01, 95% CI (−0.77 to 0.76), *p* = 0.985, *I*^2^ = 0%) (Figure 4d). A sensitivity analysis for HbA1c showed that the exclusion of Ramos (2021) [26] reduced the level of heterogeneity (*I*^2^ = 29.8%, *p* = 0.201) (Supplementary Figure S10). The level of heterogeneity in insulin was resolved (*I*^2^ = 0%) with the exclusion of Kazzi (2021) [27] (Supplementary Figure S11).

### 3.7. Liver Enzymes

**Figure 5:**
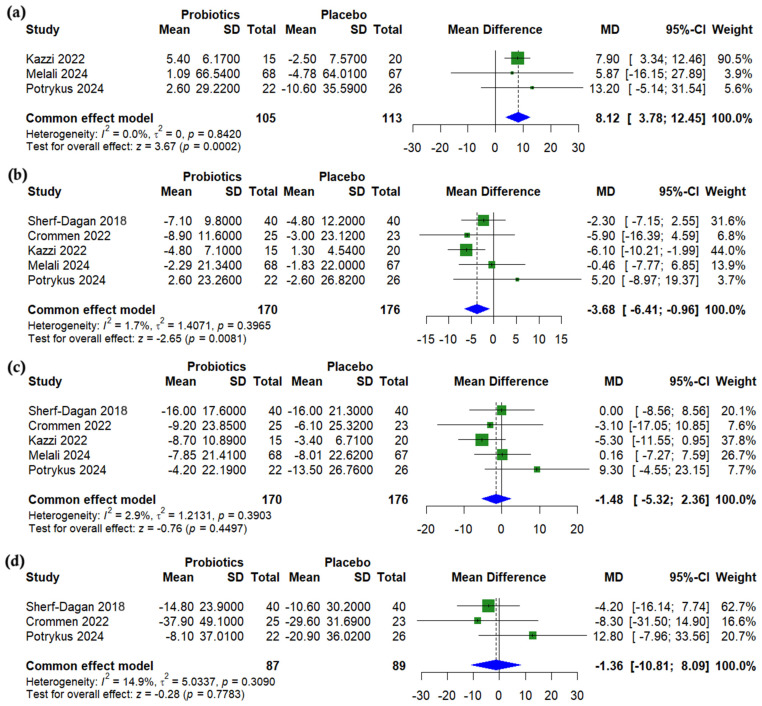
Forest plots of liver enzymes (change from baseline): (**a**) Alkaline phosphatase (ALP), U/L, (**b**) Aspartate transaminase (AST), U/L, (**c**) Alanine transaminase (ALT), U/L, (**d**) Gamma glutamyl transferase (GGT), U/L [24,25,27,29,31].

Compared to a placebo, probiotics significantly increased alkaline phosphatase (ALP) levels following bariatric surgery (MD = 8.12 U/L, 95% CI (3.78 to 12.45), *p* < 0.001, *I*^2^ = 0%) (Figure 5a). Aspartate aminotransferase (AST) was significantly lower in the probiotics group (MD = −3.68 U/L, 95% CI (−6.41 to −0.96), *p* = 0.0081, *I*^2^ = 1.7%) (Figure 5b), while no significant differences were observed for alanine aminotransferase (ALT) (MD = −1.48 U/L, 95% CI (−5.32 to 2.36), *p* = 0.45, *I*^2^ = 2.9%) (Figure 5c) or gamma-glutamyl transferase (GGT) (MD = −1.36 U/L, 95% CI (−10.81 to 8.09), *p* = 0.778, *I*^2^ = 14.9%) (Figure 5d).

### 3.8. Nutritional Outcomes

**Figure 6:**
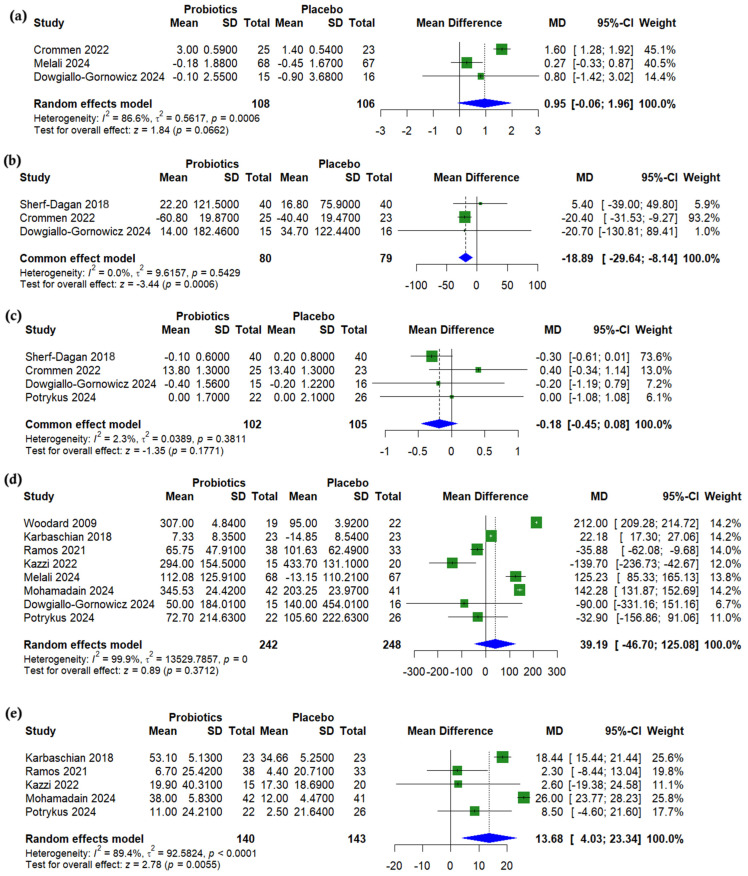
Forest plots of nutritional outcomes (change from baseline): (**a**) Albumin, g/dL, (**b**) Ferritin, µg/L, (**c**) Hemoglobin, g/dL, (**d**) Vitamin B12, pmol/L, (**e**) Vitamin D, ng/mL [19,21,22,23,24,25,26,27,29,31].

Albumin levels were slightly higher with probiotics compared to placebo, but the difference was not statistically significant (MD = 0.95 µmol/L, 95% CI (−0.06 to 1.96), *p* = 0.066, *I*^2^ = 86.6%) (Figure 6a). Ferritin levels were significantly lower with probiotic use (MD = −18.89 µg/L, 95% CI (−29.64 to −8.14), *p* < 0.001, *I*^2^ = 0.0%) (Figure 6b), while no significant differences were observed in hemoglobin (MD = −0.18 g/dL, 95% CI (−0.45 to 0.08), *p* = 0.177, *I*^2^ = 2.2%) (Figure 6c) or vitamin B12 levels (MD = 39.19 nmol/L, 95% CI (−46.70 to 125.08), *p* = 0.371, *I*^2^ = 99.9%) (Figure 6d). In contrast, vitamin D levels were significantly higher in the probiotics group (MD = 13.68 pg/mL, 95% CI (4.03 to 23.34), *p* = 0.005, *I*^2^ = 89.4%) (Figure 6e). A sensitivity analysis revealed that the heterogeneity in both vitamin B12 and vitamin D levels could not be resolved by omitting individual studies (Supplementary Figures S12 and S13). For albumin, a sensitivity analysis showed that omitting either Crommen 2022 [25] or Melali 2024 [24] resolved the heterogeneity, which decreased from 86.6% to 0% (*p* = 0.354 and 0.554, respectively) (Supplementary Figure S14).

### 3.9. Subgroup Analysis

MGB surgery was consistently associated with significant benefits, including greater excess weight loss (MD = 10.48, 95% CI: 3.15 to 17.81, *I*^2^ = 0%), lower total cholesterol (MD = −11.25, 95% CI: −22.03 to −0.46), and reduced triglycerides (MD = −20.57, 95% CI: −39.15 to −1.98). RYGB significantly decreased HbA1c (MD = −0.40, 95% CI: −0.65 to −0.14, *I*^2^ = 79%). Perioperative probiotic use was linked to significant reductions in waist circumference (MD = 6.99, 95% CI: 2.5 to 11.47, *I*^2^ = 0%), triglycerides, and total cholesterol, with a statistically significant subgroup effect for vitamin D (P for interaction = 0.004). Postoperative use was also effective in lowering HbA1c (MD = −0.22, 95% CI: −0.41 to −0.04, *I*^2^ = 63%) and triglycerides (MD = −17.47, 95% CI: −33.39 to −1.55). Comparator type influenced BMI and vitamin D outcomes, with studies using starch as comparators favoring probiotics for BMI reduction (MD = 1.02, 95% CI: 0.91 to 1.12, *I*^2^ = 0%) and increased vitamin D (MD = 15.86, 95% CI: 7.99 to 23.74, *I*^2^ = 91%). A detailed subgroup analysis is demonstrated in Supplementary Figures S15–S22.

### 3.10. Synbiotic-Specific Pooled Effects

Across the available evidence, synbiotic supplementation after bariatric surgery shows no clear benefit for anthropometric endpoints; pooled effects were statistically non-significant for BMI (+0.70 kg m^−2^), waist circumference (+0.77 cm), excess weight loss (+5.0%), and total weight loss (+1.3%), all with confidence intervals spanning clinically trivial gains and moderate-to-high heterogeneity in most cases. Cardiometabolic markers likewise revealed null findings: total cholesterol (+0.77 mg dL^−1^) and triglycerides (−16.5 mg dL^−1^) both showed no effect. Glycemic control showed a modest but statistically significant HbA1c reduction (−0.27 percentage points), yet this estimate derives from a single small trial. Vitamin D was the only biomarker to improve significantly, rising by 18.4 ng mL^−1^, again driven by a lone study (Supplementary Table S2).

## 4. Discussion

This updated meta-analysis supports that probiotic supplementation post-bariatric surgery offers modest but clinically relevant improvements in several cardiometabolic diseases and nutritional markers, with notable effects on BMI, triglycerides, HbA1c, AST, and vitamin D. By synthesizing evidence from 13 RCTs, including five new studies since the last meta-analysis, this review clarifies prior inconsistencies and introduces novel insights and future lead points through subgroup analyses by surgery type, the timing of the intervention, country, comparator, and probiotic formulation, providing a more in-depth view of the cases in which probiotics are the most effective and those in which they may fall short.

In contrast to Wang et al., who reported a small, non-significant increase in BMI with probiotic use after bariatric surgery, our updated meta-analysis demonstrates a significant reduction in BMI (MD = −0.67 kg/m^2^) following probiotic supplementation, as well as a significant decrease in HbA1c (−0.19%). Vitamin D (+13.68 pg/mL) and ferritin (−18.89 µg/L) also reached statistical significance in this update, adding nutritional context that was not evident previously. The observed increase in alkaline phosphatase (ALP, +8.12 U/L) also emerges as an unexpected and previously unreported signal related to liver function.

One of the most consistent findings was reduced triglyceride levels (MD = −16.56 mg/dL), significantly exceeding the minimal clinically important difference (MCID) threshold of 7.97 mg/dL [34]. This observation is strongly supported by a recent trial sequential analysis (TSA) by Chen and Hung (2025) [35], which demonstrated sufficient cumulative evidence for the triglyceride-lowering effect of probiotics after bariatric surgery. Individual trials contributing to this finding include Ramos et al. (2021) [26], who reported a highly significant drop in triglycerides (*p* < 0.001) with Lactobacillus acidophilus and Bifidobacterium lactis, and Crommen et al. (2022) [25], who found a similar benefit using a multistrain mixture in mini-gastric bypass (MGB) patients. In contrast, Potrykus et al. (2024) [31] reported no significant change in triglycerides; however, in this study, probiotics were administered only preoperatively.

Improvements in glycemic control were modest, with a pooled HbA1c reduction of −0.19%. These findings align with Melali et al. (2024) [24], who reported a greater decrease in HbA1c in the probiotics group both 3 and 6 months post-RYGB, and with Mohamadain et al. (2024) [19], who observed improved glycemic parameters, although the magnitude of change could not be precisely assessed due to missing baseline data. The modest magnitude of change in our analysis is likely due to the short follow-up periods in most trials. Yet, these results indicate a potential metabolic benefit of post-bariatric probiotics beyond weight loss. Furthermore, the subgroup analyses revealed that the most favorable outcomes were achieved with synbiotic formulations and when probiotic administration was initiated postoperatively, supporting the importance that timing and composition play in maximizing probiotic efficacy [36].

Although fasting insulin levels showed a modest, non-significant increase (+3.00 µU/mL) in the probiotic group, this change was not accompanied by alterations in HOMA-IR or fasting glucose. While not conclusive, such a pattern may suggest subtle shifts in insulin dynamics, possibly reflecting early compensatory mechanisms during metabolic recovery after bariatric surgery [37]. Probiotics are known to influence incretin pathways, gut permeability, and low-grade inflammation, all of which can modulate insulin dynamics [38]. However, given the short follow-up durations (1–6 months) in most included studies, the clinical relevance of this insulin trend remains uncertain and warrants further investigation over longer time frames.

Probiotic supplementation also showed a significant positive impact on vitamin D levels (MD = +13.68 pg/mL), approaching the minimal clinically significant difference of 18.9 pg/mL [39]. While suggesting a potentially meaningful effect, the clinical impact of these findings remains inconclusive and demands further investigation. The substantial heterogeneity observed across studies suggests these effects may be influenced by confounding variables such as seasonal exposure, baseline deficiency, or regional dietary habits [40].

Interestingly, probiotics were associated with a statistically significant increase in alkaline phosphatase (ALP), contrasting the otherwise favorable impact of probiotics on liver enzymes such as AST. While ALP is often thought of in the context of hepatobiliary function, it is also a sensitive marker of bone turnover and vitamin D status [41]. This rise in ALP could suggest subclinical micronutrient shifts, such as secondary hyperparathyroidism in response to calcium or vitamin D malabsorption, both of which are common after bariatric surgery [42]. Alternatively, it could reflect increased bone remodeling, which has been observed in the postoperative period as part of metabolic adaptation [43]. This increase occurred despite an accompanying rise in serum vitamin D levels, suggesting that ALP changes may not simply reflect deficiency but possibly a complex interplay between gut-mediated immune signaling, hepatic metabolism, and skeletal response. None of the included trials linked the ALP rise to adverse clinical outcomes, but this observation needs closer scrutiny, especially in longer-term studies in which bone health and liver function remain critical.

An interesting finding in this meta-analysis was the significant reduction in AST levels following probiotic supplementation without a corresponding change in ALT. While ALT is more liver-specific, AST is found in multiple tissues, including muscle, gut, and heart tissues, suggesting that the observed improvement may reflect broader systemic or metabolic effects rather than direct hepatic recovery alone [44]. This pattern could point to reduced inflammation, improved mitochondrial function, or the modulation of gut–liver axis dynamics rather than direct hepatocellular healing [45]. The absence of change in ALT, GGT, or validated liver fibrosis scores in most of the studies limits conclusions about direct hepatic benefits, yet it underscores the need for future trials incorporating liver-specific imaging or biomarkers to better clarify the hepatic versus systemic contributions of probiotics in post-bariatric patients.

Although the reduction in BMI was statistically significant (−0.99 kg/m^2^), its clinical impact remains modest. Greater weight-related benefits were observed in trials incorporating behavioral components, such as Ghafouri-Taleghani et al. (2024) [20], in which patients received dietary counseling and cognitive behavioral therapy. Similarly, Woodard et al. (2009) [21] showed a higher %EWL at 3 and 6 months in the probiotic group, though this effect diminished over time. These findings suggest that probiotics may be more effective when integrated with complementary interventions or in specific clinical scenarios such as postoperative weight regain.

An unexpected finding was decreased ferritin levels (MD = −18.89 µg/L). While this may reflect reduced inflammation, a potentially beneficial effect, low ferritin levels post-surgery also raise concerns about iron depletion. Similar effects were observed in two trials by Crommen et al. (2022) [25] and Dowgiallo-Gornowicz et al. (2024) [23], though neither clearly distinguished whether the decline was indicative of deficiencies or therapeutic benefits. These findings underscore the need for more detailed micronutrient monitoring in future studies evaluating the impact of probiotics post-bariatric surgery.

Subgroup analyses revealed that patients undergoing mini-gastric bypass (MGB) benefited the most from probiotics, with greater triglycerides, total cholesterol, and %EWL improvements. These benefits may be related to the distinct microbial shifts induced by MGB’s longer bypass limb, as suggested by Kular et al. (2018) [46]. The timing of the intervention also proved critical; probiotics administered perioperatively (before and after surgery) yielded more consistent metabolic benefits compared to preoperative-only protocols, a pattern also reported by Mohamadain et al. (2024) [19] and Karbaschian et al. (2018) [22].

Despite the strengths of these updated findings, several limitations persist. The included studies vary widely in probiotic strains, dosages, and follow-up durations, and some also involved synbiotic formulations, potentially introducing additional variability due to the adjunctive role of prebiotics. Most trials were limited to 1–6 months and often lacked reporting on key factors known to influence probiotic efficacy, such as adherence, dietary intake, or baseline microbiota composition. Also, because only one or two small synbiotic trials reported most outcomes, the effect estimates were imprecise, preventing firm conclusions about any added benefit over probiotics alone. Finally, while publication bias was detected in the BMI analysis, it remains unclear whether this reflects the true underrepresentation of null results or the influence of small sample sizes and a heterogeneous study design.

## 5. Conclusions

In summary, this updated meta-analysis reinforces the potential role of probiotics/synbiotics as a safe and modestly effective adjunct in post-bariatric care, particularly for improving triglycerides and glycemic control, as well as selecting nutritional outcomes. While not yet transformative, probiotics may support metabolic recovery in a population prone to dysbiosis, inflammation, and nutrient malabsorption. Future studies should focus on refining probiotic formulations and identifying patients most likely to benefit. In the meantime, probiotics/synbiotics represent a promising, low-risk strategy worth considering in the complex journey of metabolic surgery recovery.

## Figures and Tables

**Table 1:** Study characteristics.

Study ID	Country	Total Participants	Procedure	Probiotic Type and Content	Probiotic Dose	Placebo Details	Reported Outcomes of Interest	Follow-Up
Dowgiallo-Gornowicz 2024 [23]	Poland	31	LSG	*Lactobacillus plantarum* AMT14 5 × 10^8^ CFU g^−1^, *Bifidobacterium animalis* AMT30 1 × 10^10^ CFU g^−1^, *Bifidobacterium breve* AMT32 1 × 10^10^ CFU g^−1^	Daily	Starch	The variables assessed include gastrointestinal symptoms (stool frequency, constipation occurrence, ease of defecation, and bowel movement completeness), anthropometric measurements (BMI, %TWL, %EWL, and waist circumference), biochemical parameters (hemoglobin, ferritin, albumin, protein, glycated hemoglobin, cholesterol, triglycerides, and vitamin B12), and stool microbiota composition (Enterococcus faecalis, Enterococcus faecium, and Clostridium perfringens).	1 months
Melali 2024 [24]	Iran	135	RYGB	*Lactobacillus rhamnosus*, *Lactobacillus casei*, *Lactobacillus bulgaricus*, *Lactobacillus acidophilus*, *Bifidobacterium breve*, *Bifidobacterium longum*, *Streptococcus thermophilus* (dose not reported)	N/A	N/A	The variables assessed include anthropometric measurements (BMI, %TWL, %EWL, and weight), glycemic parameters (FBS and HbA1c), lipid profile (cholesterol, HDL, and LDL), liver enzymes (AST, ALT, and ALK-P), coagulation markers (PT, PTT, and INR), vitamins and minerals (vitamin B12, calcium, sodium, potassium, phosphorus, magnesium, and zinc), renal function markers (BUN and creatinine), and GI symptoms evaluated via the GIQLI questionnaire.	6 months
Mohamadain 2024 [19]	Egypt	83	LSG	1 × 10^9^ CFU capsule^−1^ lyophilised *Lactobacillus acidophilus* + *Bifidobacterium animalis* subsp. *lactis*; prebiotic inulin + oligofructose	BID	Inactive starch capsules	The primary outcome measured was anthropometric parameters including %EWL, BMI, weight, and WC. Secondary outcomes included lipid profile (cholesterol, triglycerides, and LDL), glycemic control (HbA1c), and serum vitamins (vitamin D [25-hydroxyvitamin D3] and vitamin B12).	4 months (4 weeks Presurgery to 12 weeks postsurgery
Potrykus 2024 [31]	Poland	48	LSG or OAGB	*Bifidobacterium bifidum* W23, *Bifidobacterium lactis* W51 & W52, *Lactobacillus acidophilus* W37, *Levilactobacillus brevis* W63, *Lacticaseibacillus casei* W56, *Ligilactobacillus salivarius* W24, *Lactococcus lactis* W19 & W58; total 2 × 10^9^ CFU day^−1^	Four capsules daily with meals (two capsules in themorning and two capsules in the evening)	Maize starch and maltodextrin from maize	The variables assessed include anthropometric measurements (weight, BMI, %WL, %EWL, and %EBMIL), glycemic parameters (glucose, insulin, HbA1c, and HOMA-IR), lipid profile (TG, HDL, LDL, and total cholesterol), liver enzymes (LDH, ALT, AST, GGT, and ALP), vitamins and minerals (vitamin D, vitamin B12, folic acid, iron, and hemoglobin), and postoperative complications (classified according to the Clavien–Dindo scale).	6 months
Ghafouri-Taleghani 2024 [20]	Iran	41	N/A (Bariatric surgery)	Per capsule: *Lactobacillus acidophilus* 1.8 × 10^9^ CFU, *Bifidobacterium bifidum* 1.8 × 10^9^ CFU, *Bifidobacterium lactis* 1.8 × 10^9^ CFU, *Bifidobacterium longum* 1.8 × 10^9^ CFU, *Lactobacillus reuteri* 1 × 10^9^ CFU, *Lactobacillus rhamnosus* 1 × 10^9^ CFU; excipients: magnesium stearate, maltodextrin	Two capsules daily	The placebo capsule contains 300 mg of starch.	The variables assessed include anthropometric measurements (weight, BMI, WC, WHR, PBF, MBF, MM, and %TWL), eating behavior scores (uncontrolled eating, cognitive restriction, emotional eating, and food addiction symptoms), serum biomarkers (leptin, oxytocin, and serotonin), dietary intake and physical activity (energy, carbohydrate, protein, fat, fiber intake, and MET), and biochemical parameters (ALT, AST, GGT, plasma glucose, insulin, HbA1c, HOMA-IR, TC, TG, LDL, HDL, IL-6, CRP, and ferritin).	3 months
Carlos 2022 [30]	Brazil	71	RYGB	5 × 10^9^ CFU tablet^−1^ *Lactobacillus acidophilus* + *Bifidobacterium lactis*	1 tablet/day	Inert manipulated tablet	The variables assessed include anthropometric measurements (weight and BMI), eating behavior scores (BES score and YFAS symptoms), and food addiction prevalence.	3 months
Crommen 2022 [25]	Germany	48	MGB	Multistrain powder 15 × 10^9^ CFU (4 g) containing *Lactobacillus acidophilus*, *Bifidobacterium breve*, *Bifidobacterium longum*, *Lactobacillus delbrueckii* subsp. *bulgaricus*, *Lactobacillus belveticus*, *Lactobacillus plantarum*, *Lactobacillus rhamnosus*, *Lactobacillus casei*, *Lactococcus lactis* subsp. *lactis*, *Streptococcus thermophilus* + 3.9 g micronutrient mix	Daily	Micronutrient mixture and a placebo powder	The variables assessed include anthropometric measurements (body mass, BMI, body fat mass, body fat-free mass, waist circumference, and visceral adiposity index), biochemical parameters (serum ALT, AST, GGT, insulin, HbA1c, HOMA-IR, TC, TG, LDL, HDL, IL-6, CRP, ferritin, hemoglobin, albumin, and total protein), liver function indices (fatty liver index, NAFLD fibrosis score, AST/ALT ratio, GGT/ALT ratio, and AST/platelet ratio), metabolic parameters (plasma glucose), and vital signs (blood pressure and heart rate).	3 months
Sherf-Dagan 2018 [29]	Israel	100	LSG	25 × 10^9^ CFU capsule^−1^ mix: *Lactobacillus acidophilus*, *Lactobacillus rhamnosus*, *Lactobacillus casei*, *Lactobacillus paracasei*, *Lactobacillus plantarum*, *Lactococcus lactis*, *Bifidobacterium bifidum*, *Bifidobacterium breve*, *Bifidobacterium longum*, *Bifidobacterium infantis*, *Streptococcus thermophilus*	2 capsules/d	No supplement	Outcomes included fatty liver assessed by the hepatorenal index and abdominal ultrasound; liver stiffness measured by shear wave elastography; anthropometric measurements (weight, height, BMI, waist circumference, hip circumference, and excess weight loss), biochemical parameters (lipid profile, C-reactive protein, glucose, hemoglobin A1c, liver enzymes, insulin, ferritin, blood count, leptin, adiponectin, cytokeratin-18, TNF-α, IL-6, IL-10, and bile acids), and microbiota composition evaluated via 16S rRNA sequencing.	3 months
Karbaschian 2018 [22]	Iran	46	OAGB	Powder (w/38.5 mg FOS) per g: *Lactobacillus casei* 3.5 × 10^9^ CFU, *Lactobacillus rhamnosus* 7.5 × 10^8^ CFU, *Streptococcus thermophilus* 1 × 10^8^ CFU, *Bifidobacterium breve* 1 × 10^10^ CFU, *Lactobacillus acidophilus* 1 × 10^9^ CFU, *Bifidobacterium longum* 3.5 × 10^9^ CFU, *Lactobacillus bulgaricus* 1 × 10^8^ CFU	Daily	Maltodextrin daily	The primary outcome of the study was a significant reduction in inflammatory factor concentrations in serum. Secondary outcomes included anthropometric measurements (weight, height, waist circumference, hip circumference, and BMI), glycemic indices (plasma glucose, insulin, HOMA-IR, and QUICKI), lipid profile (total cholesterol, triglycerides, HDL, and LDL), nutrient and vitamin levels (vitamin B12, folate, and 25-hydroxyvitamin D3), and other biochemical markers, such as homocysteine.	4 months 4 weeks pre-surgery to 12 weeks post-surgery
Kazzi 2021 [27]	USA	35	LSG	4.5 × 10^9^ CFU capsule^−1^ *Bacillus coagulans* with galactomannans (300 mg)	1 capsule/d	600 mg of calcium citrate yields 126 mg of elemental calcium. 1 capsule/day	The variables assessed include biochemical parameters, such as HbA1c, TC, LDL, HDL, TG, ALT, AST, ALP, TSH, insulin, B12, vitamin D, CRP, and fasting glucose. Additionally, anthropometric and clinical measures included SBP, DBP, % wt. loss, BMI, and % EWL.	3 months
Woodard 2009 [21]	USA	41	RYGB	*Lactobacillus* spp. (strain and dose not specified)	1 capsule/day. Each capsule contains 2.4 billion live cells	No supplementation	H2 levels, which were indicative of bacterial overgrowth, GI-related quality of life (GIQoL), serologies, weight loss, %EWL, and vitamin B12.	6 months
Ramos 2021 [26]	Brazil	101	RYGB	5 × 10^9^ CFU tablet^−1^ *Lactobacillus acidophilus* + *Bifidobacterium lactis*	1 tablet/d	Inert manipulated tablet consisting of starch and 190 mg lactose.	The variables assessed include anthropometric measurements (weight, BMI, WC, body fat, % EWL, and lean body mass), glycemic indices (FBS, HbA1c, insulin, HOMA-IR, and QUICKI), lipid profile (TC, TG, HDL, and LDL), and vitamin and biochemical markers (vitamin B12, 25-OH vitamin D3, and serum folate).	3 months
Ramos 2022 [28]	Brazil	29	RYGB	5 × 10^9^ CFU tablet^−1^ *Lactobacillus acidophilus* and 5 × 10^9^ CFU tablet^−1^ *Bifidobacterium lactis*	2 tablets/d	Inert manipulated tablet consisting of starch and 190 mg lactose.	The variables assessed include anthropometric measurements (%EWL, BMI, and weight), glycemic indices (glucose, β-hydroxybutyrate [BHB]), lipid profile, plasma metabolites (trimethylamine-N-oxide [TMAO], alanine, acetate, lactate, lipids, and acetoacetate), and branched-chain amino acids (BCAAs) (valine, leucine, and isoleucine).	3 months

**Table 2:** Baseline study data.

Study	Patients, n. (%)		Age (Mean ± SD)		Male Gender, n. (%)		Weight, kg. (Mean ± SD)		BMI (Mean ± SD)		HbA1c, % (Mean ± SD)		Smokers, n. (%)		Diabetes, n. (%)		Hypertension, n. (%)		Dyslipidemia, n. (%)		Hypothyroidism, n. (%)		Fatty Liver n. (%)	
	Probiotics	Placebo	Probiotics	Placebo	Probiotics	Placebo	Probiotics	Placebo	Probiotics	Placebo	Probiotics	Placebo	Probiotics	Placebo	Probiotics	Placebo	Probiotics	Placebo	Probiotics	Placebo	Probiotics	Placebo	Probiotics	Placebo
Dowgiallo-Gornowicz 2024 [23]	15 (48.4%)	16 (51.6%)	40.9 ± 10.6	40.9 ± 11.9	N/A	N/A	NA	NA	43.2 ± 4.7	40.1 ± 3.8	5.7 ± 0.3	6.0 ± 0.8	NA	NA	NA	NA	NA	NA	NA	NA	NA	NA	NA	NA
Melali 2024 [24]	68 (50.4%)	67 (49.6%)	33.28 ± 9.18	31.83 ± 8.62	19 (27.9%)	20 (29.8%)	125.33 ± 5.02	125.95 ± 4.38	45.98 ± 3.92	46.50 ± 4.57	5.98 ± 0.42	6.05 ± 0.18	NA	NA	NA	NA	NA	NA	NA	NA	NA	NA	NA	NA
Mohamadain 2024 [19]	42 (50.6%)	41 (49.4%)	42.72 ± 11.35	40.10 ± 9.17	7 (16.77%)	5 (12.19%)	123.14 ± 13.10	119.61 ± 12.83	43.85 ± 5.42	44.61 ± 4.35	6.15 ± 1.03	6.34 ± 1.15	NA	NA	7 (16.76%)	9 (21.95%)	11 (26.19%)	13 (31.71%)	24 (57.14%)	29 (70.73%)	3 (7.14%)	4 (9.76%)	NA	NA
Potrykus 2024 [31]	22 (45.8%)	26 (54.2%)	41.0 ± 11.2	42.2 ± 11.6	9 (40.9%)	6 (23.1%)	138.3 ± 27.1	135.9 ± 21.8	46.2 ± 6.2	45.9 ± 5.0	6.1 ± 1.3%	5.7 ± 0.7%	1 (4.5%)	7 (26.9%)	6 (27.3%)	6 (23.1%)	12 (54.5%)	14 (53.8%)	15 (68.2%)	17 (65.4%)	7 (31.8%)	9 (34.6%)	19 (86.4%)	21 (80.8%)
Ghafouri-Taleghani 2024 [20]	20 (48.8%)	21 (51.2%)	39.10 ± 6.96	39.09 ± 8.36	4 (20%)	3 (14.3%)	93.87 ± 15.24	94.93 ± 18.63	33.87 ± 4.14	34.21 ± 4.82	NA	NA	4 (20%)	4 (19%)	NA	NA	NA	NA	NA	NA	NA	NA	NA	NA
Carlos 2022 [30]	38 (53.5%)	33 (46.5%)	40.21 ± 11.25	12.7%	113.61 ± 23.21	111.21 ± 17.57	42.84 ± 5.40	43.51 ± 5.51	NA	NA	NA	NA	NA	NA	NA	NA	NA	NA	NA	NA	NA	NA		
Crommen 2022 [25]	25 (52.1%)	23 (47.9%)	40 ± 11	41 ± 9	6 (24%)	4 (17%)	127 ± 16.2	124 ± 16.9	44.3 ± 3.0	43.2 ± 3.4	5.8 ± 1.0	5.6 ± 0.7	NA	NA	7 (28%)	4 (17%)	12 (48%)	5 (22%)	NA	NA	NA	NA	25 (100%)	23 (100%)
Sherf-Dagan 2018 [29]	50 (50%)	50 (50%)	41.9 ± 9.0	41.8 ± 10.6	NA	NA	NA	NA	42.1 ± 5.0	42.5 ± 4.4	NA	NA	NA	NA	7 (14%)	6 (12%)	NA	NA	NA	NA	NA	NA	50 (100%)	50 (100%)
Karbaschian 2018 [22]	23 (50%)	23 (50%)	32.35 ± 6.88	36.95 ± 11.00	0%	0%	120.04 ± 15.10	119.34 ± 15.83	44.59 ± 4.30	44.95 ± 4.52	NA	NA	2 (8.7%)	0 (0%)	3 (13.0%)	3 (13.6%)	6 (26.1%)	8 (36.4%)	NA	NA	NA	NA	NA	NA
Kazzi 2021 [27]	15 (42.8%)	20 (57.2%)	49.3 ± 13.7	46.6 ± 11.6	3 (20%)	4 (25%)	NA	NA	43.0 ± 7.0	49.2 ± 8.3	6.0 ± 0.7%	6.0 ± 0.6%	7 (46.7%)	4 (20%)	3 (20%)	8 (40%)	9 (60%)	16 (80%)	2 (13.3%)	5 (25%)	NA	NA	NA	NA
Woodard 2009 [21]	19 (46.3%)	22 (53.7%)	48.6	41.2	3 (15.8%)	2 (9.1%)	125.4	139.0	45.7	49.6	NA	NA	NA	NA	10 (52.6%)	4 (18.2%)	11 (57.9%)	13 (59.1%)	NA	NA	NA	NA	NA	NA
Ramos 2021 [26]	51 (50.5%)	50 (49.5%)	37.1 ± 11.1	43.8 ± 10.4	4 (10.5%)	5 (15.2%)	122.24 ± 23.7	120.36 ± 20.7	43.95 ± 5.43	44.31 ± 5.3	~5.1 ± 1.41	~5.3% ± 1.67	0 (0%)	1 (3.0%)	6 (15.8%)	7 (21.2%)	11 (28.9%)	21 (63.6%)	27 (71.1%)	18 (54.4%)	NA	NA	26 (68.4%)	25 (75.8%)
Ramos 2022 [28]	13 (44.8%)	16 (55.2%)	37.00 ± 12.88	47.00 ± 8.47	2 (15.4%)	3 (18.8%)	NA	NA	41.94 ± 6.47	45.13 ± 6.75	NA	NA	0 (0%)	1 (6.2%)	1 (7.7%)	4 (25.0%)	4 (30.8%)	10 (62.5%)	9 (69.2%)	5 (31.2%)	NA	NA	12 (92.3%)	10 (62.5%)

BMI: body mass index, HbA1c: glycated hemoglobin. Smoking reflects both current and former smoking status. Categorical data are presented in frequency (percentage), and continuous data are presented in mean ± SD.

## Data Availability

Data are available on reasonable request.

## Supplementary Materials

The following supporting information can be downloaded at: https://www.mdpi.com/article/10.3390/nu17132193/s1, Supplementary Table S1. Search strategy; Supplementary Table S2. Synbiotic-specific pooled effects; Supplementary Figure S1. Risk of bias assessment using RoB2; Supplementary Figure S2. Sensitivity Analysis of BMI; Supplementary Figure S3. Sensitivity Analysis of Waist Circumference (WC); Supplementary Figure S4. Sensitivity Analysis of Total Weight Loss % (TWL%); Supplementary Figure S5. Meta-regression analysis of BMI depending on age; Supplementary Figure S6. Meta-regression analysis of BMI depending on baseline BMI; Supplementary Figure S7. Meta-regression analysis of TWL depending on age; Supplementary Figure S8. Meta-regression analysis of TWL depending on baseline; Supplementary Figure S9. Funnel plot of BMI; Supplementary Figure S10. Funnel plot of TWL; Supplementary Figure S11. Sensitivity Analysis of HbA1c %; Supplementary Figure S12. Sensitivity Analysis of insulin; Supplementary Figure S13. Sensitivity Analysis of Vitamin B12; Supplementary Figure S14: Sensitivity Analysis of Vitamin D; Supplementary Figure S15: Sensitivity Analysis of Albumin; Supplementary Figure S16. Subgroup Analysis of BMI; Supplementary Figure S17. Subgroup Analysis of Waist Circumference (WC); Supplementary Figure S18. Subgroup Analysis of Excessive Weight Loss % (EWL%); Supplementary Figure S19. Subgroup Analysis of Total Weight Loss % (TWL%); Supplementary Figure S20. Subgroup Analysis of Total Cholesterol (TC); Supplementary Figure S21. Subgroup Analysis of Triglycerides; Supplementary Figure S22. Subgroup Analysis of HbA1c %; Supplementary Figure S23. Subgroup Analysis of Vitamin D.

## Abbreviation List

The following abbreviations are used throughout this manuscript: ALP (alkaline phosphatase), ALT (alanine aminotransferase), AST (aspartate aminotransferase), ATP (anti-tachycardia pacing), BHB (β-hydroxybutyrate), BES (Binge Eating Scale), BMI (body mass index), CFU (colony-forming unit), CI (confidence interval), CRP (C-reactive protein), DBP (diastolic blood pressure), EWL (excess weight loss), FBS (fasting blood sugar), GGT (gamma-glutamyl transferase), GI (gastrointestinal), GIQLI (Gastrointestinal Quality of Life Index), HDL (high-density lipoprotein), HOMA-IR (homeostatic model assessment for insulin resistance), ITT (intention-to-treat), LDL (low-density lipoprotein), LSG (laparoscopic sleeve gastrectomy), MCID (minimal clinically important difference), MGB (mini-gastric bypass), OAGB (one-anastomosis gastric bypass), PP (per-protocol), PRISMA (Preferred Reporting Items for Systematic Reviews and Meta-Analyses), PROSPERO (International Prospective Register of Systematic Reviews), QUICKI (Quantitative Insulin Sensitivity Check Index), RCT (randomized controlled trial), ROB2 (Risk of Bias 2 tool), RR (risk ratio), RYGB (Roux-en-Y gastric bypass), SBP (systolic blood pressure), SD (standard deviation), TSA (trial sequential analysis), TWL (total weight loss), WC (waist circumference), WHR (waist-to-hip ratio), and YFAS (Yale Food Addiction Scale).
