# Supplementary material for: Impact of Probiotic/Synbiotic Supplementation on Post-Bariatric Surgery Anthropometric and Cardiometabolic Outcomes: An Updated Systematic Review and Meta-Analysis of Randomized Controlled Trials
Source: Nutrients. 2025 Jun 30;17(13):2193. doi: 10.3390/nu17132193 (PMC12251824; doi:10.3390/nu17132193)
Supplement: Supplementary file 1 [file nutrients-17-02193-s001.zip › nutrients-3666010-supplementary.pdf]

Supplementary Table S1. Search strategy

Database	Search Query	Results
PubMed	(Probiotics OR Prebiotics OR Synbiotics OR "gut microbiota" OR "gut flora" OR "microbiome" OR "intestinal microbiota" OR "gut bacteria" OR "microbial diversity") AND (bariatric surgery OR "weight loss surgery" OR "metabolic surgery" OR "Roux-en-Y gastric bypass" OR "RYGB" OR "sleeve gastrectomy" OR "gastric sleeve" OR "biliopancreatic diversion" OR "adjustable gastric banding" OR "laparoscopic gastric bypass")	809
Scopus	TITLE-ABS-KEY ((Probiotics OR Prebiotics OR Synbiotics OR "gut microbiota" OR "gut flora" OR "microbiome" OR "intestinal microbiota" OR "gut bacteria" OR "microbial diversity") AND (bariatric surgery OR "weight loss surgery" OR "metabolic surgery" OR "Roux-en-Y gastric bypass" OR "RYGB" OR "sleeve gastrectomy" OR "gastric sleeve" OR "biliopancreatic diversion" OR "adjustable gastric banding" OR "laparoscopic gastric bypass"))	1,060
Web of science	(Probiotics OR Prebiotics OR Synbiotics OR "gut microbiota" OR "gut flora" OR "microbiome" OR "intestinal microbiota" OR "gut bacteria" OR "microbial diversity") AND (bariatric surgery OR "weight loss surgery" OR "metabolic surgery" OR "Roux-en-Y gastric bypass" OR "RYGB" OR "sleeve gastrectomy" OR "gastric sleeve" OR "biliopancreatic diversion" OR "adjustable gastric banding" OR "laparoscopic gastric bypass") (Topic)	1,136
Cochrane	(Probiotics OR Prebiotics OR Synbiotics OR "gut microbiota" OR "gut flora" OR "microbiome" OR "intestinal microbiota" OR "gut bacteria" OR "microbial diversity") AND (bariatric surgery OR "weight loss surgery" OR "metabolic surgery" OR "Roux-en-Y gastric bypass" OR "RYGB" OR "sleeve gastrectomy" OR "gastric sleeve" OR "biliopancreatic diversion" OR "adjustable gastric banding" OR "laparoscopic gastric bypass") in All Text - (Word variations have been searched)	157

Supplementary Table S2. Synbiotic-specific pooled effects

Outcome	Synbiotic trials (n)	Pooled MD	95 % CI	p-value	I^2 (%)
Body-mass index (BMI)	3	0.70	–0.13 to 1.54	0.10	66
Waist circumference (WC)	2	0.77	–3.94 to 5.48	0.75	39
Excess weight loss (%)	2	5.04	–4.16 to 14.24	0.28	84
Total weight loss (%)	3	1.27	–1.23 to 3.77	0.32	69
Total cholesterol (TC)	2	0.77	–3.94 to 5.48	0.75	39
Triglycerides (TG)	1	–16.45	–47.35 to 14.45	0.30	—
HbA1c	1	–0.27	–0.43 to –0.11	0.001	—
Vitamin D	1	18.44	15.44 to 21.44	0.00001	—

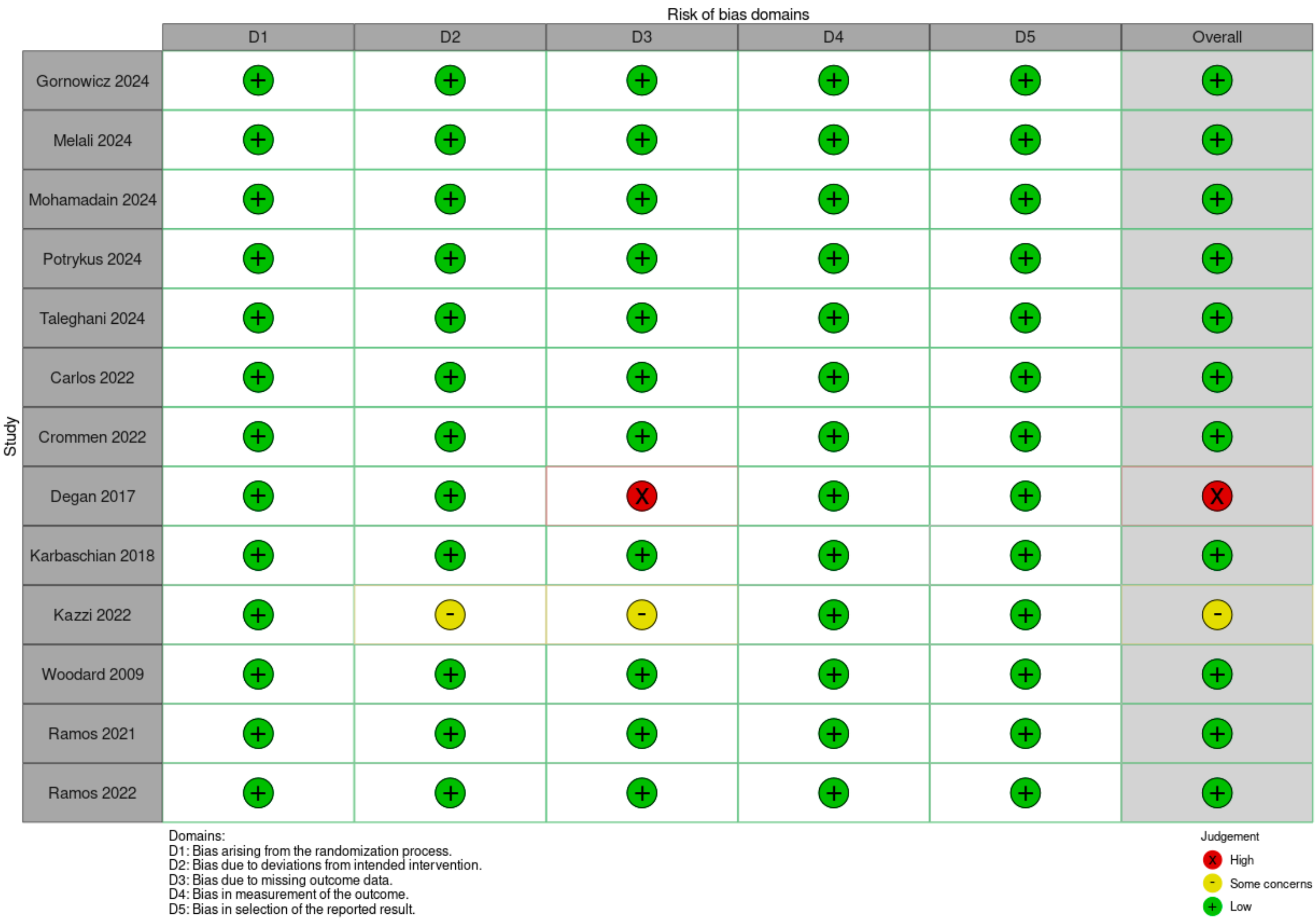

Supplementary Figure S1. Risk of bias assessment using RoB2

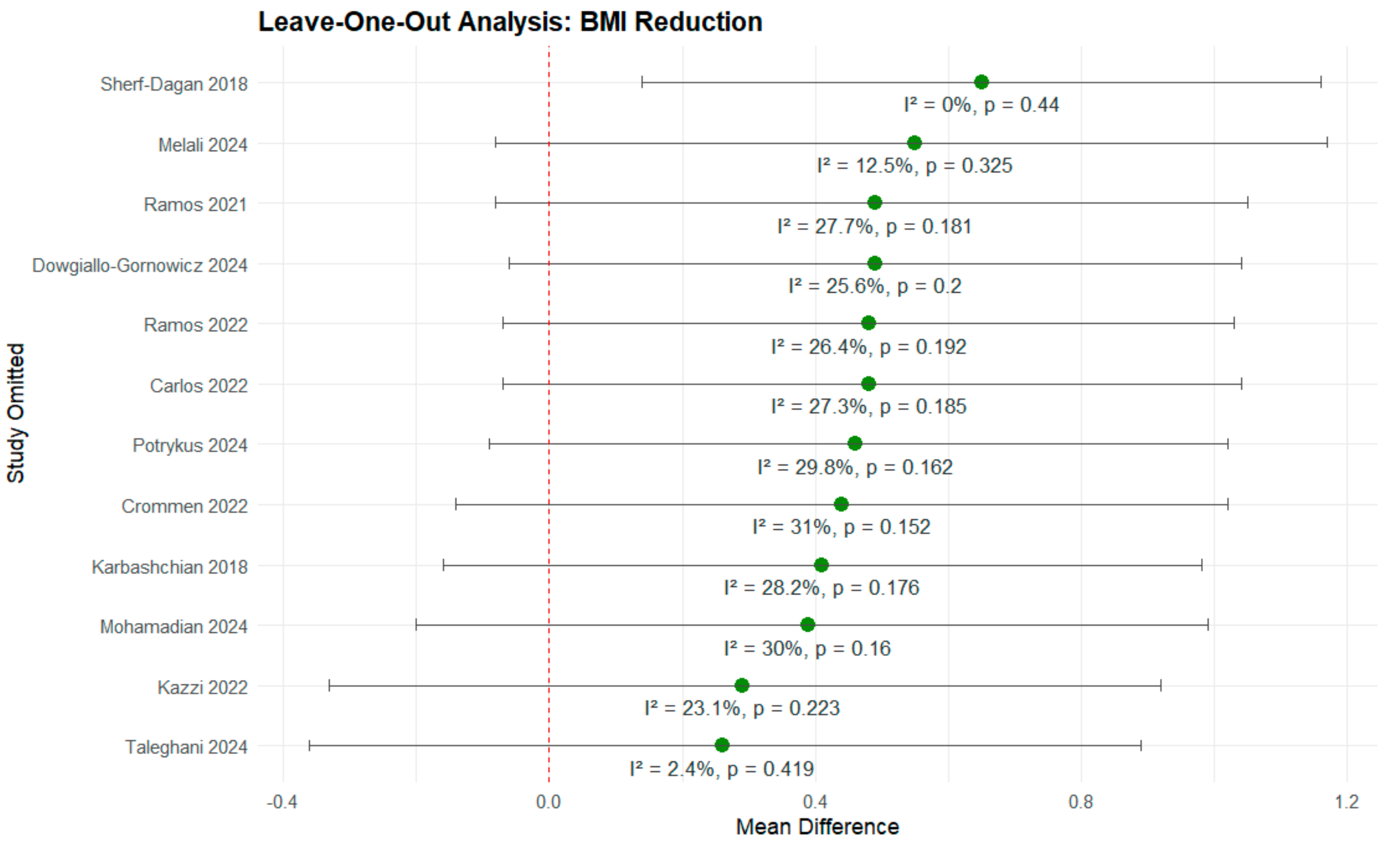

Supplementary Figure S2. Sensitivity Analysis of BMI

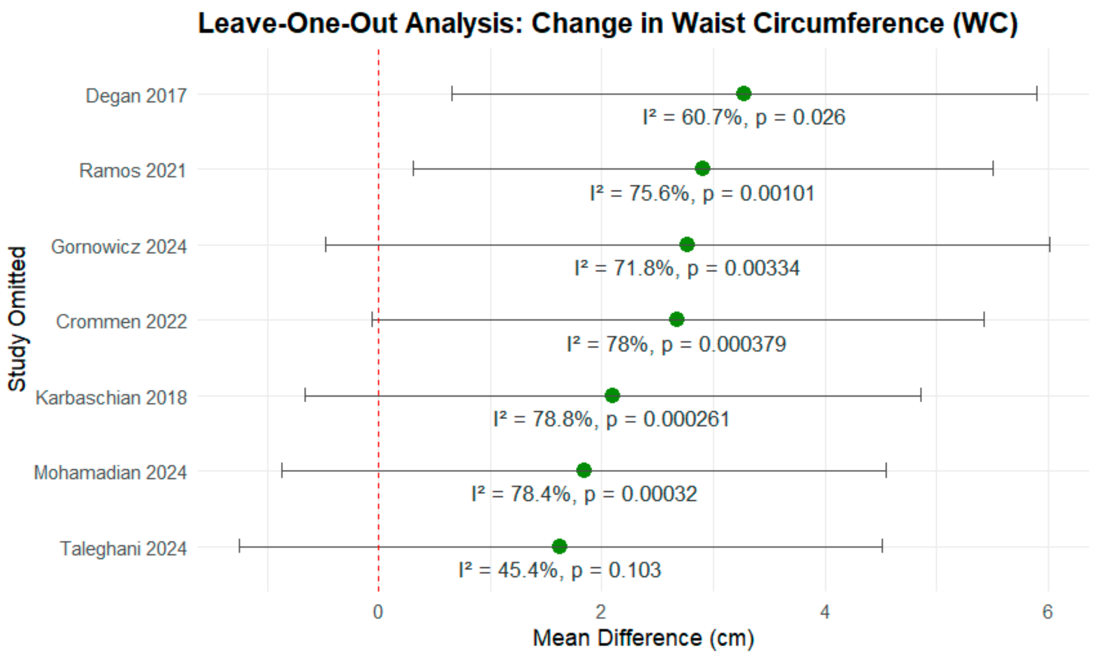

Supplementary Figure S3. Sensitivity Analysis of Waist Circumference (WC)

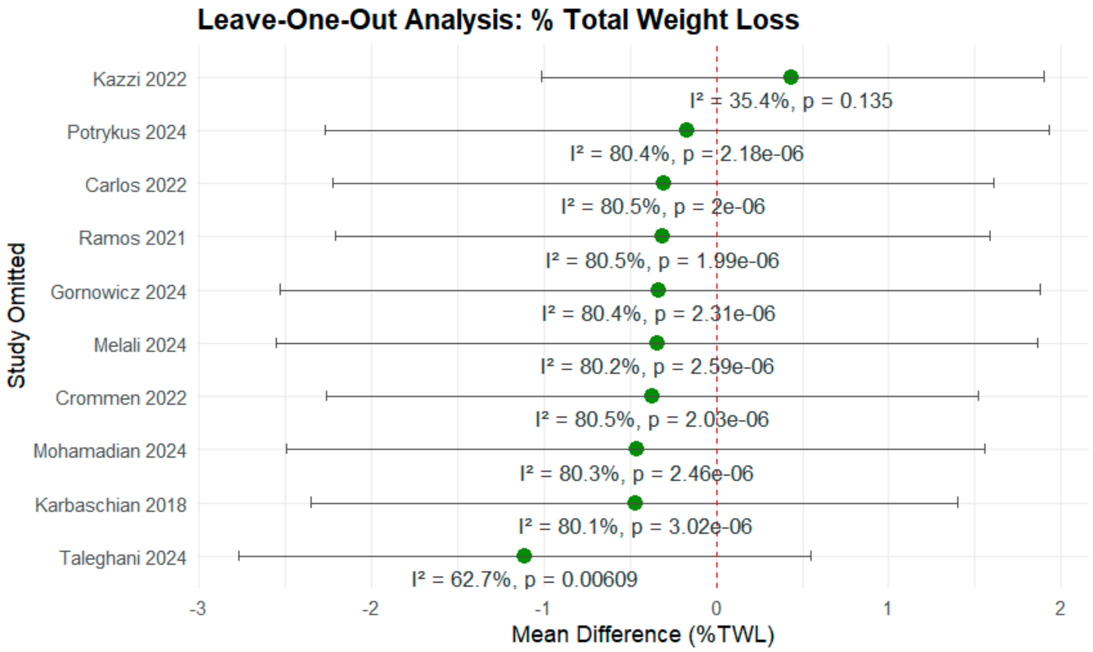

Supplementary Figure S4. Sensitivity Analysis of Total Weight Loss % (TWL%)

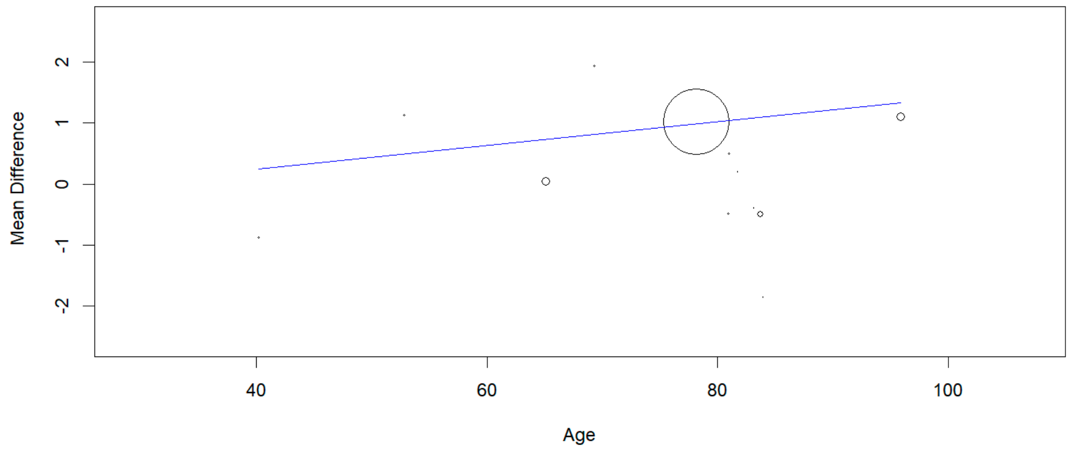

Supplementary Figure S5. Meta-regression analysis of BMI depending on age

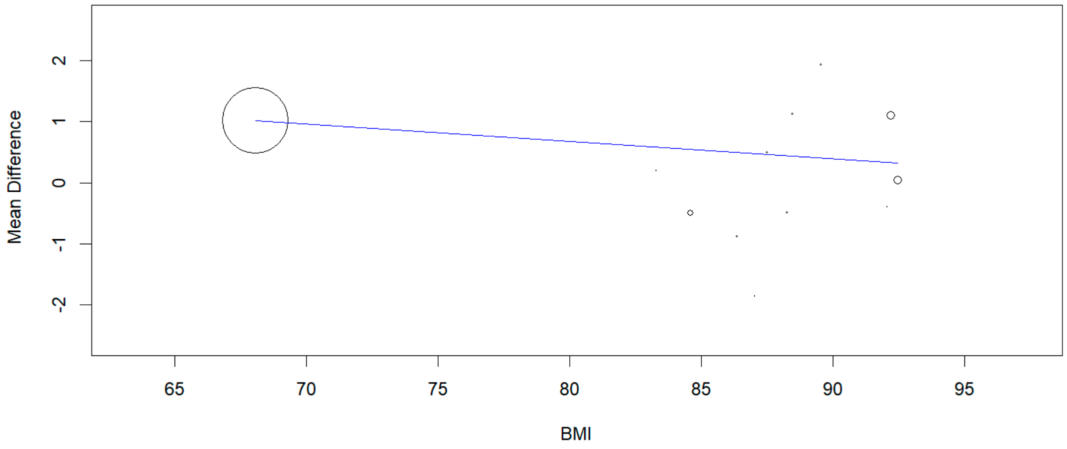

Supplementary Figure S6. Meta-regression analysis of BMI depending on baseline BMI

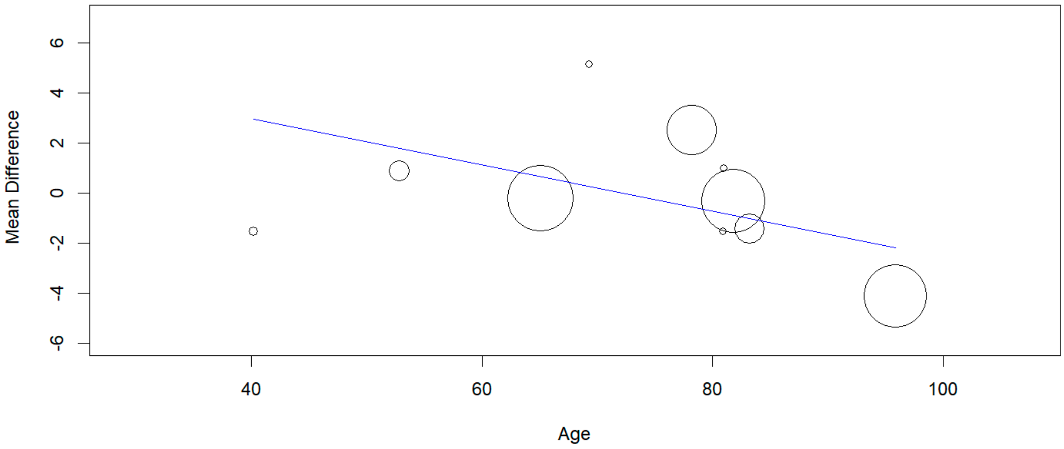

Supplementary Figure S7. Meta-regression analysis of TWL depending on age

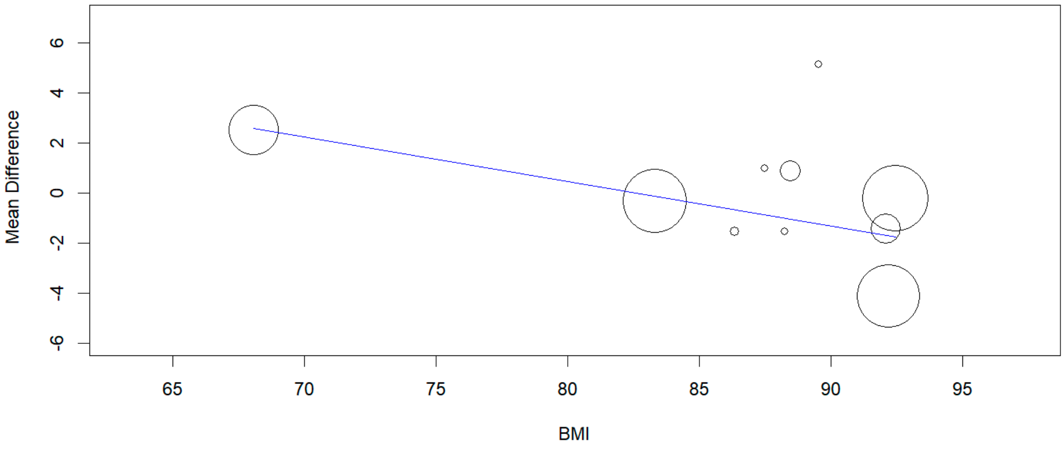

Supplementary Figure S8. Meta-regression analysis of TWL depending on baseline BMI

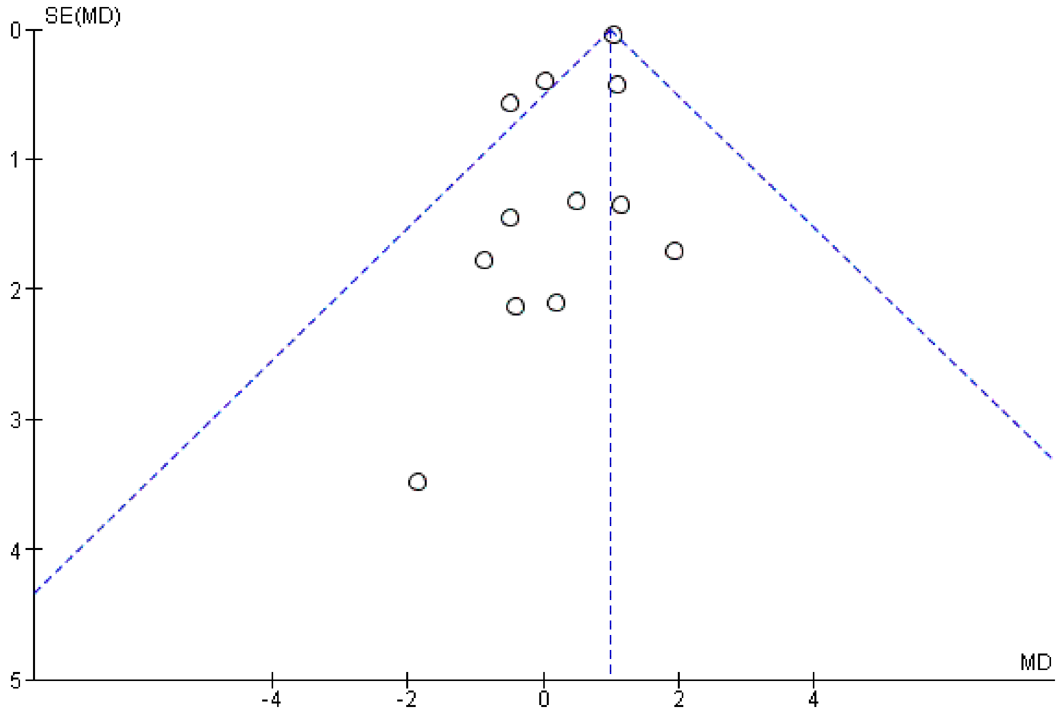

Supplementary Figure S9. Funnel plot of BMI

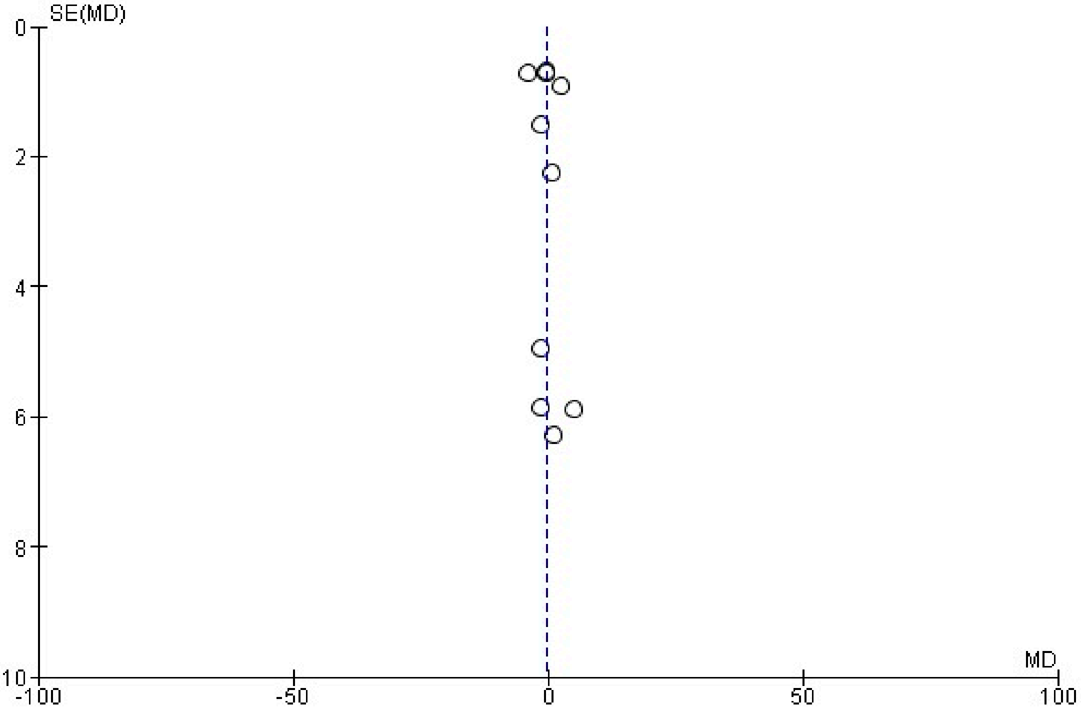

Supplementary Figure S10. Funnel plot of TWL

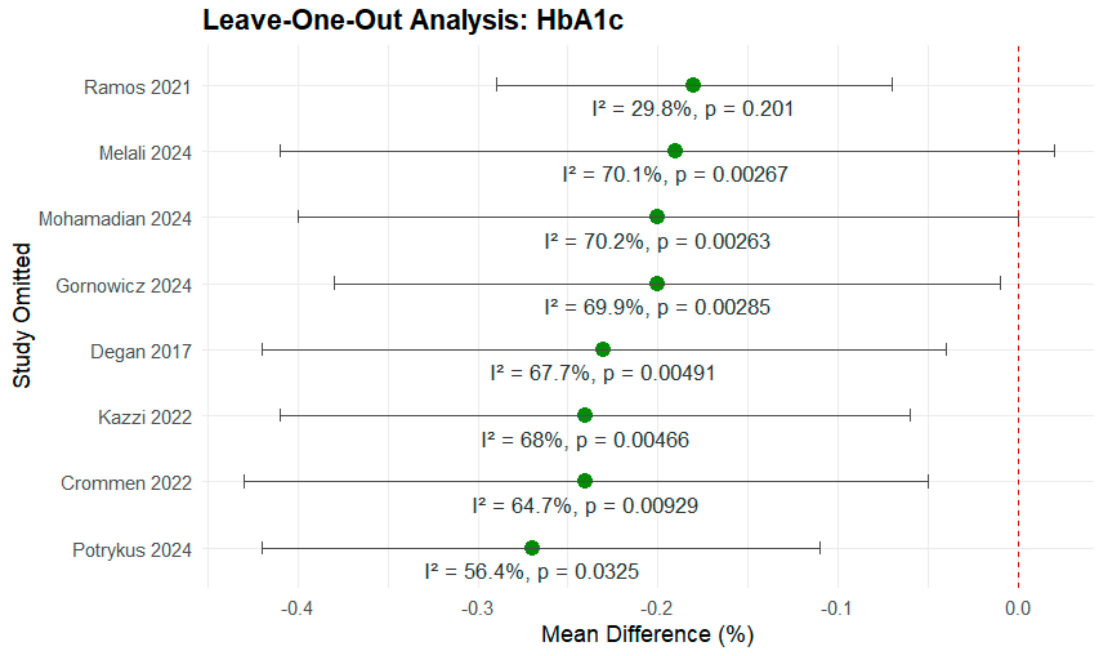

Supplementary Figure S11. Sensitivity Analysis of HbA1c %

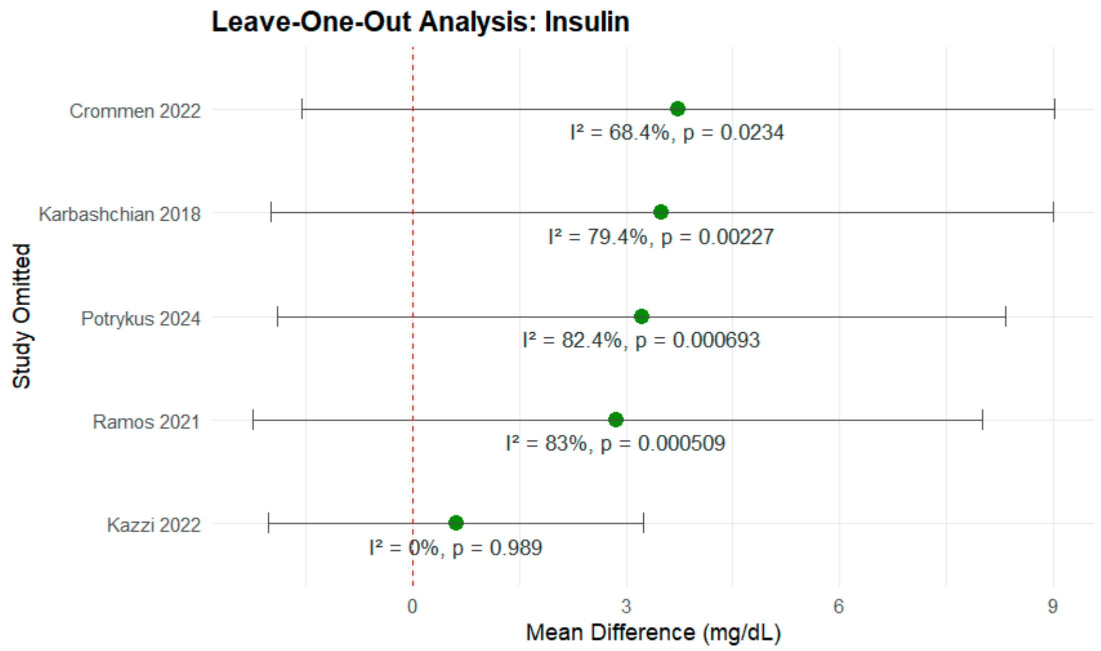

Supplementary Figure S12. Sensitivity Analysis of insulin

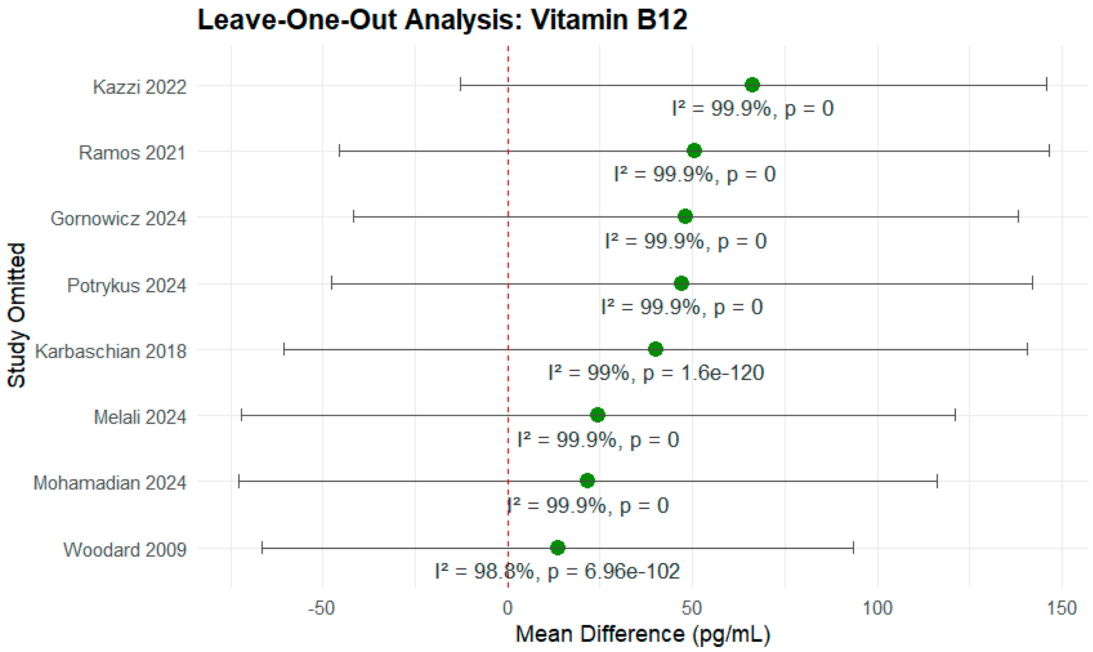

Supplementary Figure S13. Sensitivity Analysis of Vitamin B12

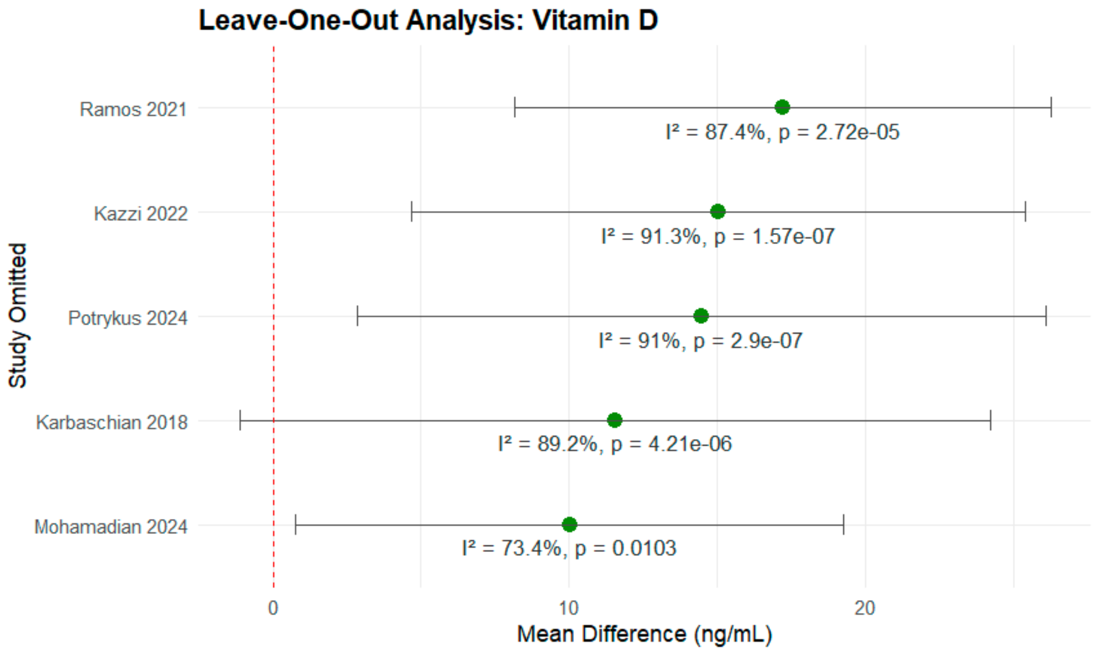

Supplementary Figure S14. Sensitivity Analysis of Vitamin D

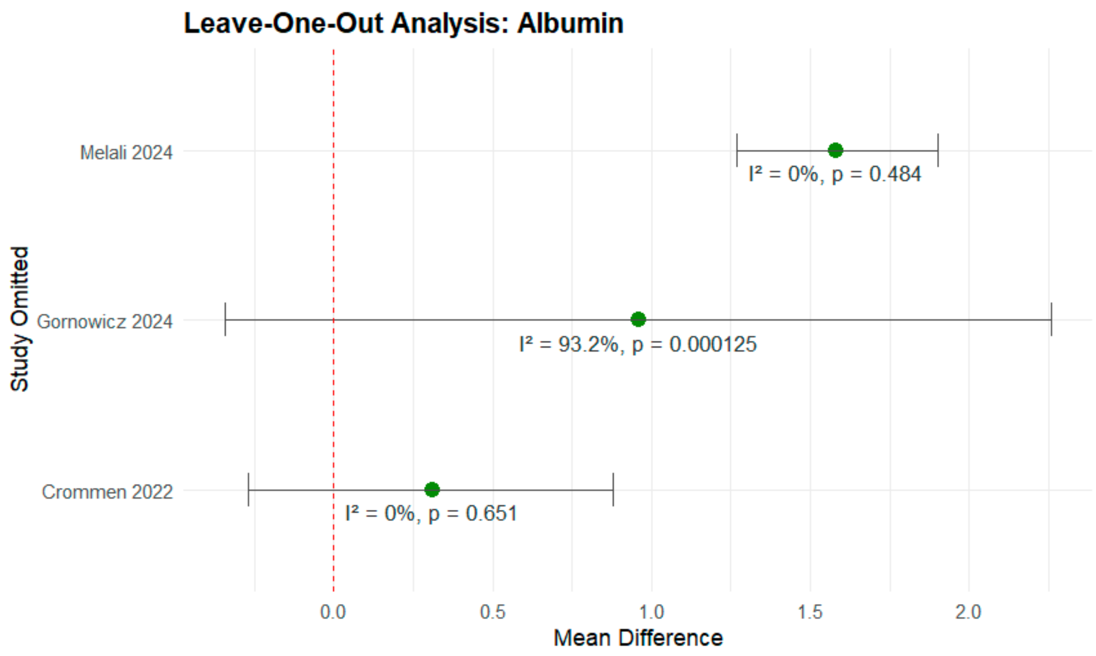

Supplementary Figure S15. Sensitivity Analysis of Albumin

Outcome	No. of studies	I²	MD (95% CI)	p	P for interaction
**Bariatric surgery type**					**0.06**
LSG	4	42	0.46 [-0.58, 1.5]	0.38	
RYGB	4	0	-0.06 [-0.81, 0.69]	0.87	
MGB	2	0	1.04 [-1.01, 3.10]	0.32	
Mixed (LSG or OAGB)	1		-0.40 [-4.59, 3.79]	0.85	
Unclear	1		1.02 [0.91, 1.13]	0.0001	
**Intervention Components**					**0.58**
Probiotics only	9	0	0.42 [-0.18, 1.01]	0.17	
Probiotics + Prebiotics	3	66	0.7 [-0.13, 1.54]	0.1	
**Time of intervention**					**0.61**
Postoperative	9	0.11	0.46 [-0.01, 1.03]	0.05	
Perioperative	2	0	1.44 [-0.63, 3.51]	0.71	
Preoperative	1		-0.4 [-4.59, 3.79]	0.85	
**Country**					**0.63**
Poland	2	0	-0.09 [-3.03, 2.84]	0.95	
Iran	3	66	0.7 [-0.13, 1.54]	0.1	
Brazil	3	0	-0.75 [-2.85, 1.34]	0.48	
Others	4	42	0.48 [-0.5, 1.46]	0.34	
**Comparator**					**0.006**
Starch	7	0	1.02 [0.91, 1.12]	0.0001	
Others	5	36	0.30 [-0.20, 0.80]	0.24	

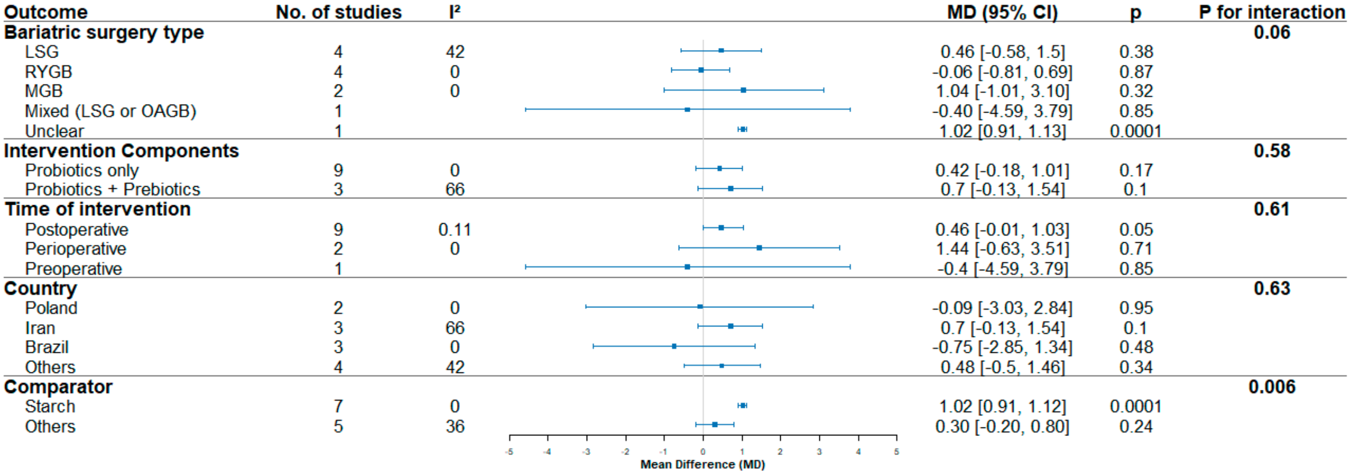

Supplementary Figure S16. Subgroup Analysis of BMI

Outcome	No. of studies	I²	MD (95% CI)	p	P for interaction
**Bariatric surgery type**					**0.4**
LSG	3	0	18.90 [-14.57, 52.36]	0.27	
RYGB	2		-4.23 [-13.35, 4.89]	0.36	
MGB	1		3.40 [-4.54, 11.34]	0.4	
Unclear	1		2.14 [1.68, 2.60]	0.0001	
**Intervention Components**					**0.1**
Probiotics only	7	0	-5.56 [-11.32, 0.21]	0.06	
Probiotics+Prebiotics	2	39	0.77 [-3.94, 5.48]	0.75	
**Time of intervention**					**0.8**
Postoperative	5	0	9.52 [-9.62, 28.66]	0.33	
Perioperative	2	0.97	6.99 [2.5, 11.47]	0.002	
**Comparator**					**0.38**
Starch	5	42	2.37 [0.37, 4.37]	0.02	
Others	2	99	24.02 [-24.58, 72.63]	0.33	

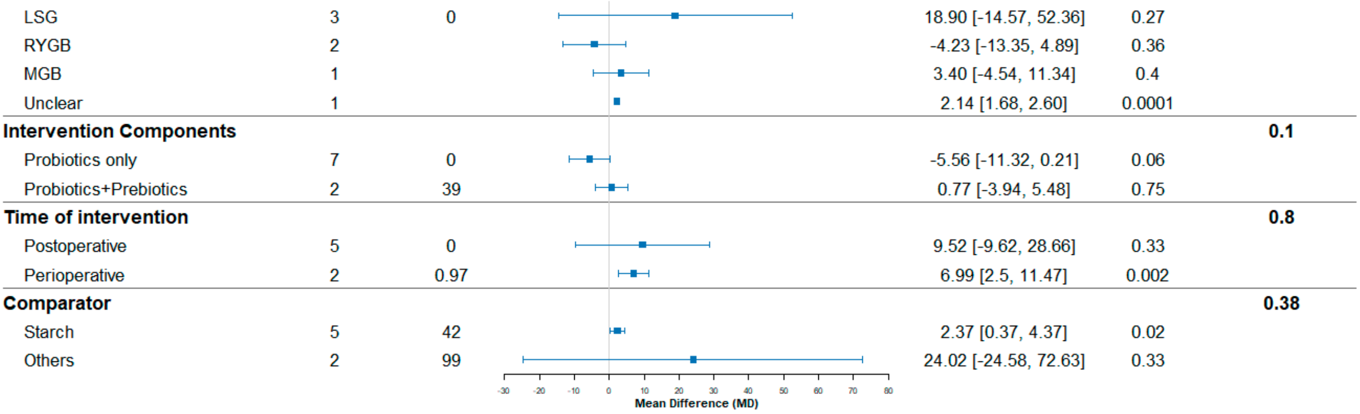

Supplementary Figure S17. Subgroup Analysis of Waist Circumference (WC)

Outcome	No. of studies	I²	MD (95% CI)	p	P for interaction
**Bariatric surgery type**					**0.007**
LSG	3	48	-0.95 [-4.79, 2.89]	0.63	
RYGB	4	68	3.17 [-0.97, 7.30]	0.13	
MGB	1		10.48 [3.15, 17.81]	0.005	
Mixed (LSG or OAGB)	1		-0.40 [-8.76, 7.96]	0.005	
**Intervention Components**					**0.42**
Probiotics only	7	64	0.99 [-2.75, 4.72]	0.6	
Probiotics + Prebiotics	2	84	5.04 [-4.16, 14.24]	0.28	
**Time of intervention**					**0.44**
Postoperative	6	71	0.89 [-2.58, 4.37]	0.61	
Perioperative	2	69	5.99 [-1.64, 13.61]	0.12	
Preoperative	1		-0.4 [-8.78, 7.96]	0.93	
**Country**					**0.27**
Poland	2	0	-2.53 [-6.37, 1.32]	0.2	
Iran	2	84	5.04 [-4.16, 14.24]	0.28	
Brazil	2	0	1.47 [-7.82, 10.75]	0.76	
Others	3	75	2.56 [-2.43, 7.55]	0.31	
**Comparator**					**0.97**
Starch	6	53	1.84 [-2.47, 6.16]	0.4	
Others	3	83	1.97 [-2.52, 6.46]	0.39	

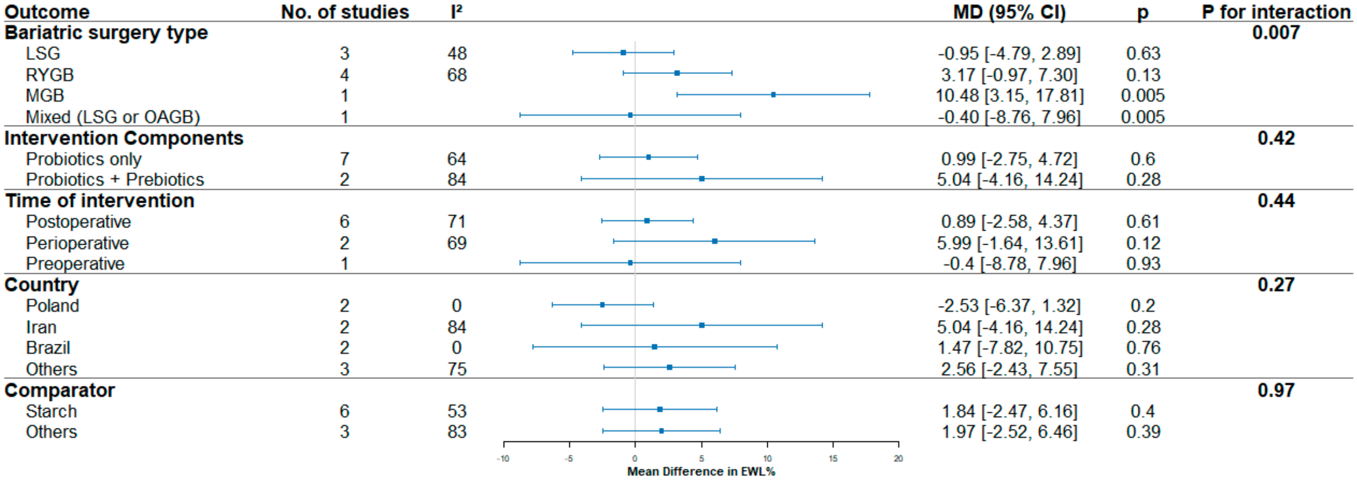

Supplementary Figure S18. Subgroup Analysis of Excessive Weight Loss % (EWL%)

Outcome	No. of studies	I²	MD (95% CI)	p	P for interaction
**Bariatric surgery type**					**0.05**
LSG	3	87	-1.48 [-4.63, 1.67]	0.36	
RYGB	3	0	-0.25 [-1.58, 1.08]	0.71	
MGB	2	0	3.22 [-5.18, 11.61]	0.45	
Mixed (LSG or OAGB)	1		-1.40 [-4.38, 1.58]	0.36	
Unclear	1		2.54 [0.76, 4.32]	0.005	
**Intervention Components**					**0.10**
Probiotics only	7	62	-1.49 [-3.58, 0.59]	0.16	
Probiotics + Prebiotics	3	69	1.27 [-1.23, 3.77]	0.32	
**Time of intervention**					**0.54**
Postoperative	7	83	-0.58 [-2.85, 1.69]	0.61	
Perioperative	2	0	1.44 [-2.66, 5.54]	0.49	
Preoperative	1		-1.4 [-4.38, 1.58]	0.36	
**Country**					**0.52**
Poland	2	0	-0.5 [-1.78, 0.77]	0.44	
Iran	3	69	1.27 [-1.23, 3.77]	0.32	
Brazil	2	0	-1.53 [-8.94, 5.88]	0.69	
Others	3	60	-1.85 [-5.97, 2.28]	0.38	
**Comparator**					**0.18**
Starch	6	42	0.58 [-1.07, 2.24]	0.49	
Others	4	80	-1.90 [-5.13, 1.33]	0.25	

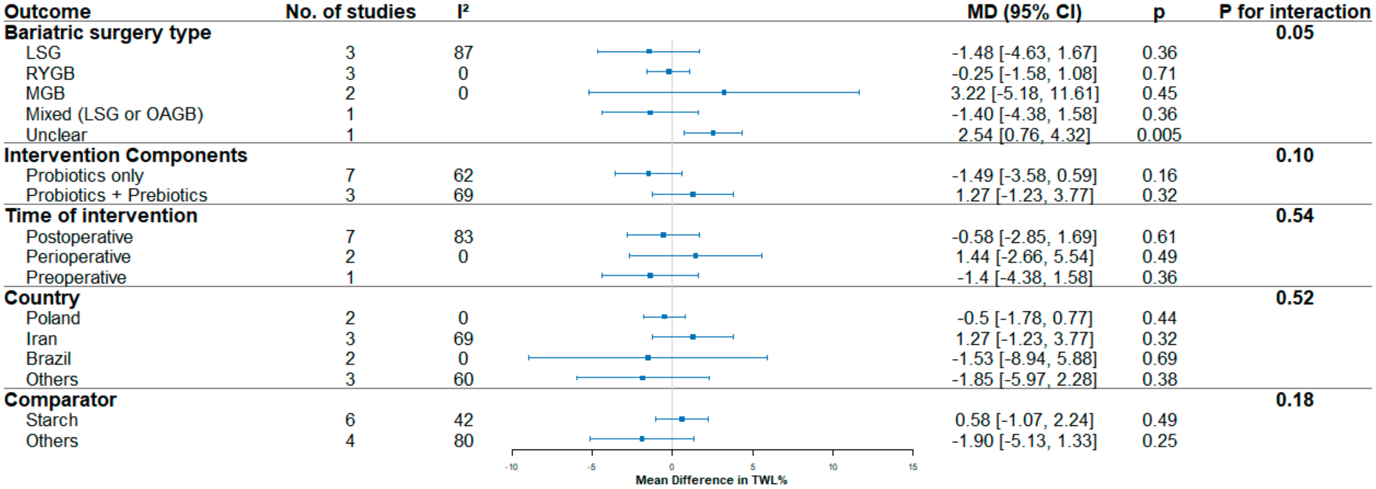

Supplementary Figure S19. Subgroup Analysis of Total Weight Loss % (TWL%)

Outcome	No. of studies	I²	MD (95% CI)	p	P for interaction
**Bariatric surgery type**					**0.1**
LSG	4	0	-4.55 [-11.11, 2.01]	0.17	
RYGB	2	19	1.30 [-3.57, 6.16]	0.6	
MGB	2	0	-11.25 [-22.03, -0.46]	0.04	
Mixed (LSGor OAGB)	1		14.30 [-16.07, 44.67]	0.36	
**Intervention Components**					**0.1**
Probiotics only	7	0	-5.56 [-11.32, 0.21]	0.06	
Probiotics+Prebiotics	2	39	0.77 [-3.94, 5.48]	0.75	
**Time of intervention**					**0.11**
Postoperative	6	0	-0.16 [-4.34, 4.02]	0.94	
Perioperative	2	0	-8.23 [-15.93, -0.53]	0.04	
Preoperative	1		14.3 [-16.07, 44.67]	0.36	
**Comparator**					**0.11**
Starch	5	0	-6.82 [-13.95, 0.30]	0.06	
Others	4	13	0.04 [-4.21, 4.28]	0.99	

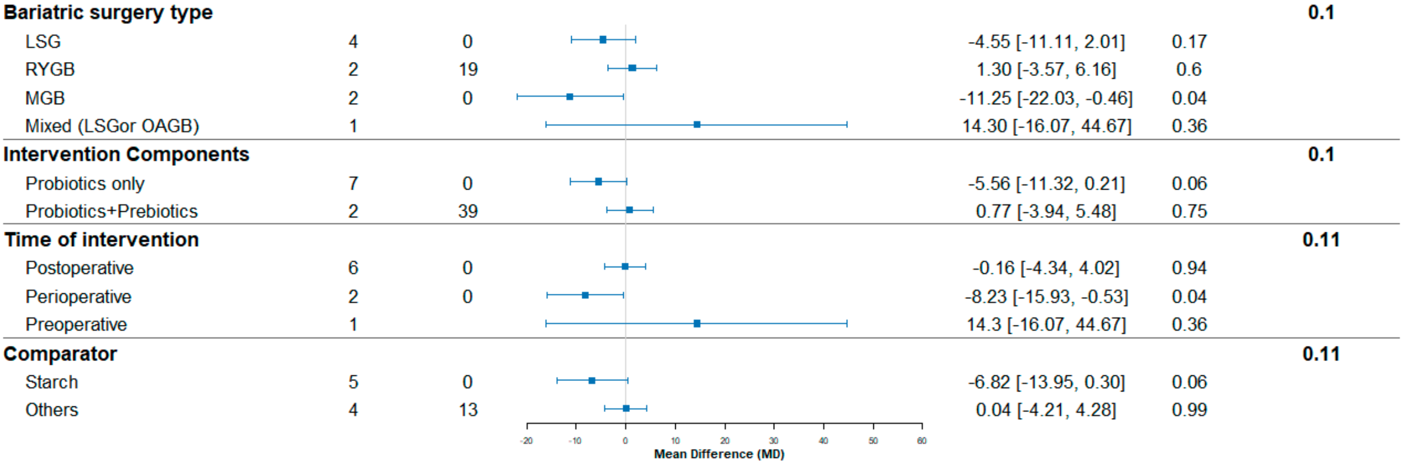

Supplementary Figure S20. Subgroup Analysis of Total Cholesterol (TC)

Outcome	No. of studies	I²	MD (95% CI)	p	P for interaction
**Bariatric surgery type**					**0.87**
LSG	4	0	-15.18 [-30.55, 0.20]	0.05	
RYGB	1		-22.09 [-88.23, 44.05]	0.51	
MGB	2	0.03	-20.57 [-39.15, -1.98]	0.03	
Mixed (LSG or OAGB)	1		1.40 [-49.24, 52.04]	0.96	
**Intervention Components**					**0.99**
Probiotics only	7	0	-16.58 [-28.8, -4.36]	0.008	
Probiotics+Prebiotics	1		-16.45 [-47.35, 14.45]	0.3	
**Time of intervention**					**0.78**
Postoperative	5	0	-17.47 [-33.39, -1.55]	0.03	
Perioperative	2	0	-17.57 [-34.69, -0.44]	0.04	
Preoperative	1		1.40 [-49.24, 52.04]	0.96	
**Comparator**					**0.57**
Starch	5	0	-13.76 [-28.74, 1.22]	0.07	
Others	3	0	-20.36 [-37.8, -2.92]	0.02	

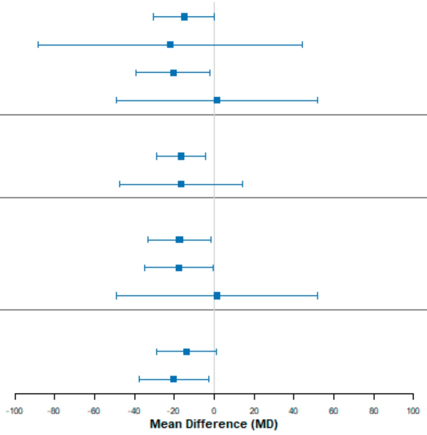

Supplementary Figure S21. Subgroup Analysis of Triglycerides

Outcome	No. of studies	I²	MD (95% CI)	p	P for interaction
**Bariatric surgery type**					**0.04**
LSG	4	0	-0.13 [-0.32, 0.05]	0.16	
RYGB	2	79	-0.4 [-0.65, -0.14]	0.002	
MGB	1		-0.1 [-0.39, 0.19]	0.5	
Mixed (LSGor OAGB)	1		0.6 [-0.09, 1.29]	0.09	
**Intervention Components**					**0.38**
Probiotics only	7	70	-0.14 [-0.38, 0.1]	0.24	
Probiotics+Prebiotics	1		-0.27 [-0.43, -0.11]	0.001	
**Time of intervention**					**0.06**
Postoperative	6	63	-0.22 [-0.41, -0.04]	0.02	
Perioperative	1		-0.3 [-0.63, 0.03]	0.08	
Preoperative	1		0.6 [-0.09, 1.29]	0.09	
**Comparator**					**0.88**
Starch	4	77	-0.16 [-0.55, 0.23]	0.42	
Others	4	0	-0.19 [-0.32, -0.07]	0.003	

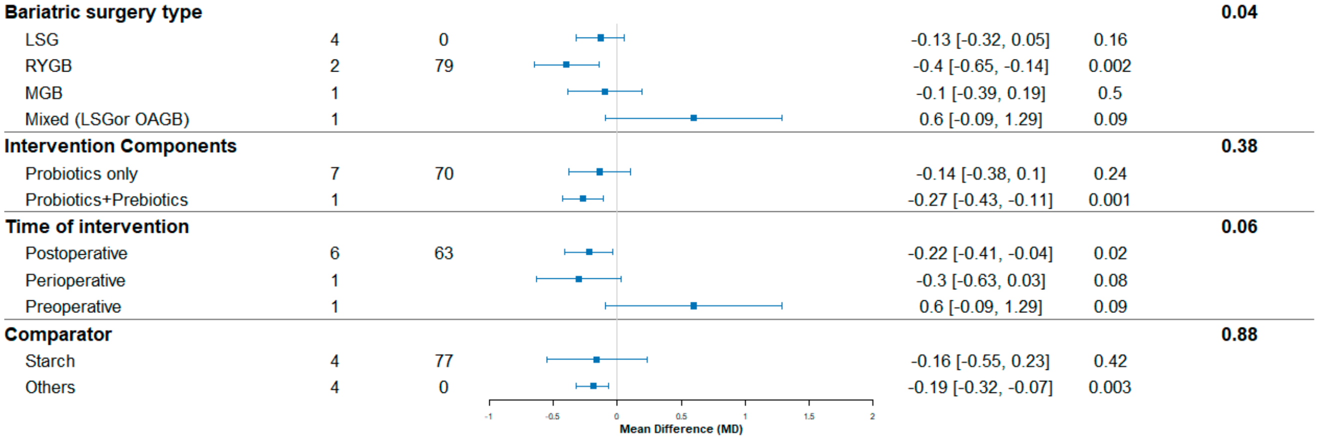

Supplementary Figure S22. Subgroup Analysis of HbA1c

Outcome	No. of studies	I²	MD (95% CI)	p	P for interaction
**Bariatric surgery type**					**0.02**
LSG	2	77	16.96 [-5.37, 39.29]	0.14	
RYGB	1		2.30 [-8.44, 13.04]	0.67	
MGB	1		18.44 [15.44, 21.44]	0.0001	
Mixed (LSGor OAGB)	1		8.50 [-4.60, 21.60]	0.2	
**Intervention Components**					**0.35**
Probiotics only	4	89	11.02 [-4.41, 26.45]	0.16	
Probiotics+Prebiotics	1		18.44 [15.44, 21.44]	0.00001	
**Time of intervention**					**0.004**
Postoperative	2	0	2.36 [-7.29, 12.0]	0.63	
Perioperative	2	94	22.29 [14.88, 29.7]	0.0001	
Preoperative	1		8.5 [-4.6, 21.6]	0.2	
**Comparator**					**0.27**
Starch	4	91	15.86 [7.99, 23.74]	0.00001	
Others	1		2.60 [-19.38, 24.58]	0.82	

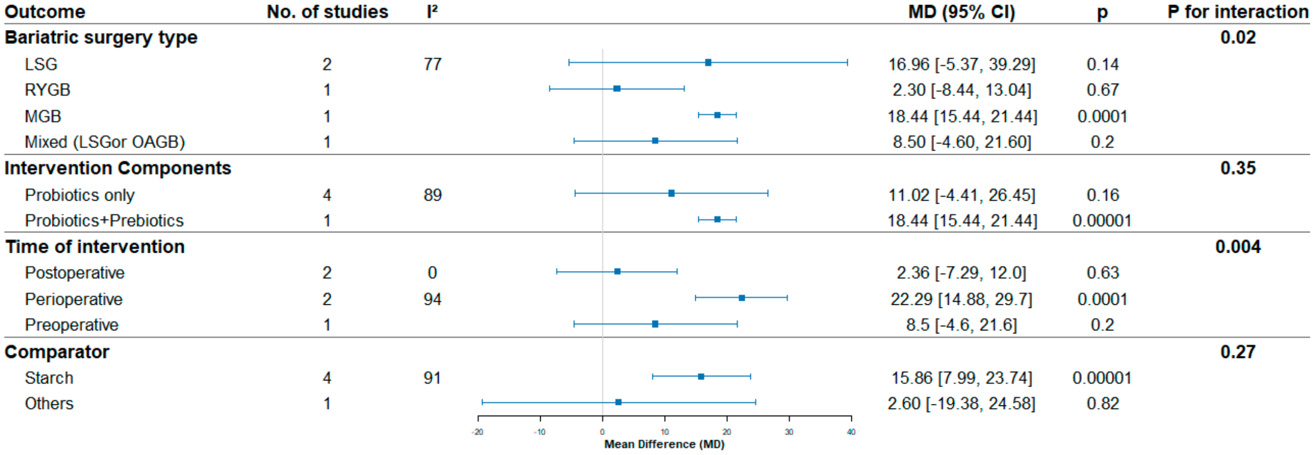

Supplementary Figure S23. Subgroup Analysis of Vitamin D
